# Epithelial and immune transcriptomic characteristics and possible regulatory mechanisms in asthma exacerbation: insights from integrated studies

**DOI:** 10.3389/fimmu.2025.1512053

**Published:** 2025-01-23

**Authors:** Ye Liu, Yue Li, Ruhao Wu, Yu Wang, Pengfei Li, Tianci Jiang, Ke Wang, Yize Liu, Zhe Cheng

**Affiliations:** Department of Respiratory and Critical Care Medicine, The First Affiliated Hospital of Zhengzhou University, Zhengzhou, He’nan, China

**Keywords:** asthma exacerbation, single-cell RNA sequencing, bulk RNA sequencing, epithelial cells, immune cells, key regulatory genes

## Abstract

**Background:**

Asthma exacerbation significantly contribute to disease mortality and result in heightened health care expenditures. This study was aimed at gaining important new insights into the heterogeneity of epithelial and immune cells and elucidating key regulatory genes involved in the pathogenesis of asthma exacerbation.

**Methods:**

Functional enrichment, pseudotime, metabolism and cell-cell communication analyses of epithelial cells and immune cells in single-cell RNA sequencing (scRNA-seq) dataset were applied. Immune infiltration analysis was performed in bulk RNA sequencing (bulk RNA-seq) dataset. Key regulatory genes were obtained by taking the intersection of the differentially expressed genes (DEGs) between control and asthma group in epithelial cells, immune cells and bulk RNA-seq data. Asthma animal and *in vitro* cell line models were established to verify the key regulatory genes expression by employing quantitative reverse transcription polymerase chain reaction (qRT-PCR).

**Results:**

ScRNA-seq analysis identified 7 epithelial subpopulations and 14 distinct immune cell types based on gene expression profiles. Further analysis demonstrated that these cells manifested high heterogeneity at the levels of functional variations, dynamics, communication patterns and metabolic changes. Notably, TMPRSS11A, TUBA1A, SCEL, ICAM4, TMPRSS11B, IGFBP2, CLC, NFAM1 and F13A1 were identified as key regulatory genes of asthma. The results of the qRT-PCR demonstrated that the 9 key regulatory genes were involved in asthma.

**Conclusions:**

We systematically explored epithelial and immune characteristics in asthma exacerbation and identified 9 key regulatory genes underlying asthma occurrence and progression, which may be valuable for providing new insights into the cellular and molecular mechanisms driving asthma exacerbations.

## 1 Introduction

Patients with asthma exacerbation bear a substantial burden of disability, economic costs, and healthcare utilization (1, 2). The clinical management of allergic asthma is challenging due to phenotypic heterogeneity, as some patients exhibit mild disease that responds well to therapy, while others suffer from severe, progressive disease that does not respond effectively to conventional treatments. This variability indicates that allergic disease populations consist of various subgroups, each with unique underlying mechanisms (3, 4). Therefore, the underlying mechanisms of asthma are still insufficiently elucidated.

Advances in genomics have made bulk RNA sequencing (bulk RNA-seq) a key tool for studying gene alterations in asthma. This methodology enables a meticulous analysis of gene expression patterns within complete cell populations during diseased conditions (5). For instance, Jiang Yong with colleagues revealed key genes and immune cell infiltration patterns associated with severe asthma progression via bulk RNA-seq analysis (6). Key genes and pathways in mild−moderate, steroid−resistant asthma or neutrophilic asthma were also identified (7–9). Nowadays, more and more researchers have realized that bulk RNA-seq are limited in resolving cellular heterogeneity in disease and single-cell RNA sequencing (scRNA-seq) analysis in asthma has increased rapidly (10). This powerful technique offers the potential to elucidate the complex interactions between diverse cell types and their roles in the pathogenesis of asthma. Jehan Alladina’s study revealed unique transcriptional programs and cell circuits by comparing allergic asthma to allergic individuals without asthma (11). Felipe A. Vieira Braga et al. reported the cellular census and intercellular communications in healthy and asthmatic airway walls, which was the most comprehensive analysis of asthma scRNA-seq data to date (12). However, the combined single-cell and bulk RNA-seq data analysis in asthma exacerbation remains an underexplored approach, which could yield deeper insights into the molecular mechanisms.

The incorporation of bioinformatics analysis techniques can synergize the advantages of single-cell and bulk RNA-seq methodologies, thereby improving the robustness of results and enhancing the depth of insights derived from the data (13). In this study, we aimed to provide a comprehensive characterization of the epithelial and immune transcriptomic landscape and potential mechanisms in asthma exacerbation pathogenesis by combining scRNA-seq and bulk RNA-seq data. By leveraging the single-cell resolution of scRNA-seq, we identified the diverse cellular subtypes, functional variations, cell-type dynamics, metabolic changes and communication netwoks that underlie the heterogeneity of epithelial and immune cells, while the incorporation of bulk RNA-seq data will enable the identification of key regulatory genes. Additionally, we further verified these key regulatory genes in the asthma model. This study will offer new insights into the cellular and molecular mechanisms driving asthma exacerbations, potentially paving the way for the development of more effective therapeutic interventions.

## 2 Materials and methods

### 2.1 Data acquisition

The scRNA-seq dataset was obtained from GSE164015 (https://www.ncbi.nlm.nih.gov/geo/query/acc.cgi?acc=GSE164015) in the Gene Expression Omnibus (GEO) database at the National Center for Biotechnology Information (NCBI) (14). Dataset GSE164015 contained 8 bronchoalveolar lavage fluid (BALF) samples collected at bronchoscopy in 4 independent participants with asthma. They were sampled 1 day after the right middle lobe was challenged with an allergen to which they were allergic (dust, mite, or cat) or the right upper lobe was challenged with diluent as a control. We took the former as the asthma group (A) and the latter as the control group (C) (14).

**Figure 1:**
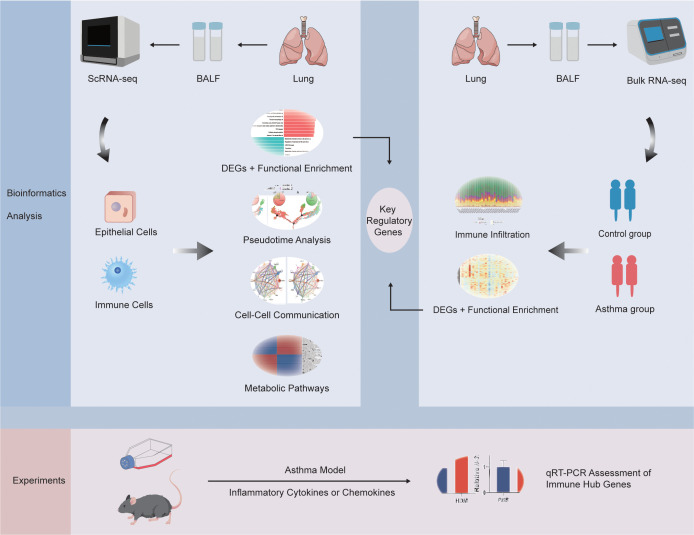
Flow diagram of the overall study design.

Because we utilized Bulk RNA-seq of BALF to intersect key regulatory genes, GSE136587 was one of the few datasets of BALF samples tested from patients with varying degrees of asthma (https://www.ncbi.nlm.nih.gov/geo/query/acc.cgi?acc=GSE136587) (15, 16). GSE136587 was based on the GPL18573 platform and included 39 BALF samples comparing healthy (6 samples), mild (17 samples) or severe asthma (16 samples) patients. We combined the data of healthy individuals as the control group (C) and patients with moderate and severe asthma as the asthma group (A) (15). The workflow of this study was demonstrated in **Figure 1**.

### 2.2 Data processing and analysis

We performed scRNA-seq analysis by transforming the raw gene expression matrix into a Seurat object using Seurat package (v4.3.0.1) of RStudio (v2023.6.0.421) (17, 18). Cells with > 200 and < 2500 features per cell, < 25% mitochondrial genes, and genes expressed in at least 3 cells were retained for further analysis. 33,388 filtered cells were selected for analysis. Each sample was characterized with 2000 highly variable genes (HVGs) through the “vst” selection method. Principal component analysis (PCA) was then employed to identify significant principal components (PCs), and the *p* value distribution was visualized using the “JackStraw” and “ScoreJackStraw” functions (19). Batch correction was conducted using the “harmony” package (v1.2.0) to mitigate batch effects due to sample identity (20). In data clustering, we used the package ‘clustree’ (v0.5.1) from Seurat (21). In brief, this method can visualize how clusters break down and display the classification results from one resolution to another, so that we can refer to which resolution is more appropriate. Cells were classified into different clusters by using “FindClusters” with 0.1 resolution (all cells), 0.1 resolution (epithelial cells), and 0.5 resolution (immune cells). The Uniform Manifold Approximation and Projection (UMAP) analysis was used for the visualization plot with the two-dimensional UMAP model (22). In each cluster, the marker genes of cell populations were identified using the “FindMarkers” or “FindAllMarkers” function with the Wilcoxon rank-sum test. We integrated this information with online databases, including the Annotation of Cell Types (ACT) Database (http://xteam.xbio.top/ACT/) (23), the CellMarker 2.0 database (http://117.50.127.228/CellMarker/index.html) (24) and Human Transcriptome Cell Atlas (HTCA) database (https://www.htcatlas.org/) (25). Ultimately, canonical markers from the existing literature were utilized to arrive at a comprehensive determination of the final cell type (11, 12, 26, 27). Based on the evaluation of cluster-specific cell markers, we clustered the data into epithelial and immune cell groups at first. Then we analyzed the epithelial cells and immune cells respectively. For a second clustering of the cell populations, the same procedures were repeated. Epithelial cells were classified into 7 different clusters and a total of 14 clusters of immune cells were defined.

In RNA-seq data, raw data was downloaded using the “GEOquery” package (v2.70.0) (28). We performed differentially expressed genes (DEGs) analysis of bulk RNA-seq data using the R package “DEseq2” (v1.42.1) (29). *P* value < 0.05 and an absolute log2FoldChange (|log2FC|) > 0.5 were considered statistically significant. Volcano and heatmap plots were drawn using the “ggplot2” package (v3.5.0) and “pheatmap” package (v1.0.12) (30, 31).

### 2.3 Heterogeneity and correlation assessment

ROGUE (v1.0) was employed using default parameter settings for recommended pipelines to effectively evaluate the purity of identified cell clusters (32). Spearman’s correlation was used to analyze the correlation between the cell types.

### 2.4 Distribution of cell types across groups

The group preference of individual cell types was evaluated by calculating the ratio of observed to expected cell numbers (Ro/e) for each cluster (33). Ro/e represents the ratio of observed cell count to expected cell count within a specific grouping of cell clusters and distinct groups. The expected cell count for each grouping was determined through the utilization of the chi-squared test.

### 2.5 Supervised analyses using genome-wide association study genes

The asthma-associated GWAS gene list was obtained from the GWAS Catalog of EMBL-EBI by searching for “asthma” (https://www.ebi.ac.uk/gwas/). This list was retrieved on December 14, 2023. Subsequently, we identified the genes that were common between our single-cell DEGs list and the asthma-associated GWAS list. Normalized average expression levels of intersecting genes were subjected to hierarchical clustering analysis, arranging genes in rows and single cells in columns.

### 2.6 Functional enrichment analysis

The “FindMarkers” function was employed to identify the DEGs for each cluster, facilitating the exploration of group functions. DEGs were identified using a cutoff of *p* value < 0.05 and |Log2FC|> 0.5. By using the “enrichKEGG” and “enrichGo” functions in the R package “clusterProfiler” (v4.10.1), the biological function analysis of DEGs was conducted to analyze the biological pathways based on Gene Ontology (GO) (http://geneontology.org/) and Kyoto Encyclopedia of Genes and Genomes (KEGG) pathway database (https://www.genome.jp/kegg/pathway.html) (34). *P* value < 0.05, adjusted using the Benjamini–Hochberg method, was established as the cut-off criterion. The enrichment results were visualized by R packages “fmsb” (v0.7.6), “enrichplot” (v1.22.0), “ggplot2” (v3.5.0), and OmicShare tools (https://www.omicshare.com/tools).

Gene set enrichment analysis (GSEA) evaluates the enrichment of genes within a set at the extremes of a ranked list (35). We used the “clusterProfiler” (v4.10.1) package and “gseKEGG” or “gseGO” function to identify GO terms and KEGG pathways. The GSEA analysis was performed according to default parameters. Furthermore, GSEA was utilized to assess the presence of significant differences in predefined gene sets between two groups. The hallmark gene set was derived from the molecular signature database (MSigDB, http://www.gsea-msigdb.org/gsea/msigdb/index.jsp) (36). Annotation clusters with *p* value <0.05 were considered statistically significant.

### 2.7 Trajectory analysis

The Monocle2 algorithm was employed to infer the differentiation trajectories of the selected clusters with the “monocle” package (v2.30.0) (37). Cells were arranged along a pseudotime trajectory using the combined set of HVGs from the cells. Low-quality cells and genes were identified and removed using the “detectGenes” and “subset” functions, respectively, with the “min_expr” parameter set to 0.1. The “differentialGeneTest” function was utilized to identify DEGs among clusters along the trajectory. For branch site differential genes analysis, the “BEAM” package (v2.0.2) was employed to identify the genes most significantly contributing to cell branching in the branch site differential genes analysis. Genes identified by the Branch Expression Analysis Modeling (BEAM) analysis with a *q-*value ≤ 0.01 were hierarchically clustered using the “plot_genes_branched_heatmap” function with num_clusters = 4. The heat map showed the first 50 critical branch differential genes. Genes from each respective hierarchical cluster were input into GO or KEGG analysis to further investigate enrichment functions.

### 2.8 Cell-cell communication analysis

CellChat (v1.6.1) with default recommended settings was employed to evaluate cell-cell interactions among various cell types (38). We loaded the CellChatDB.human database into RStudio and selected the signal path of Secreted Signaling, ECM-Receptor, and Cell-Cell Contact in the database. Cell groups with fewer than 10 cells were filtered using the “filterCommunication” function with the “min.cells” parameter. The “mergeCellChat” was used to merge the two CellChat objects so that we could further analyze the communication characteristics of the two groups.

### 2.9 Evaluation of metabolic activity at single-cell resolution

The method for analyzing the activity of metabolic pathways of individual cells within each cell population was AUCell (v1.24.0) algorithm from scMetabolism package (v0.2.1) in RStudio (39). This study utilized KEGG metabolic gene sets for analysis.

### 2.10 Immune infiltration analysis

CIBERSORTx (v0.1.0) algorithm was applied to creat a reference matrix for deconvoluting immune cell abundances in each bulk RNA-seq sample (40). Gene expression data with standard annotations were analyzed using LM22 signatures and 1000 permutations in RStudio.

### 2.11 Regulatory mechanisms of key genes and transcription factors

To explore the critical regulatory genes involved in both the exacerbation and pathogenesis of asthma, we intersected the DEGs with the most significant difference (*p* value <0.05 and |log2FC| > 0.5) between C and A in epithelial cells, immune cells, and bulk RNA-seq data. To avoid the bias effects of different statistical methods, “DEseq2” (v1.42.1) package was employed to analyze the DEGs in the two datasets. Upstream transcription factors (TFs) and miRNA of key regulated genes were predicted through the NetworkAnalyst database (https://www.networkanalyst.ca/) (41). mRNA-TFs pairs were predicted via JASPAR algorithms and miRNA-mRNA pairs were predicted through the mirTarBase v8.0 (42, 43). Finally, the mRNA-TFs-miRNA interaction network analysis was visualized using Cytoscape (v3.10.2) to explore regulatory mechanisms (44).

### 2.12 Establishment of asthma animal model

Female C57/BL6N wild-type (WT) mice (4-6weeks; weight 19-21g; n=12), purchased from Beijing Vital River Laboratory Animal Technology Co., Ltd., were kept in specific pathogen-free conditions with inrestricted access to food and water. Mice were raised at a constant temperature of 23 ± 2°C rooms. Room lighting was automatically controlled on a 12 h light/dark cycle. The mice were acclimatized for one week prior to the experiment. Mice were randomly distributed into asthma (A) and control (C) groups (n = 6 for each group). Mouse models of asthma were established as previously described (45). Briefly, 100μg house dust mite (HDM) (Dermatophagoides Pteronyssinus, Greer Laboratories, USA) in 50 μl phosphate buffered saline (PBS, Solarbio, China) were intranasally delivered to the mice in asthma group for sensitization (day 0). In control group, mice were intranasally delivered with an equal volume of PBS. Beginning 1 week after the sensitization, mice were challenged daily with 10μg HDM in 50μl PBS by intranasal administration (day 7-11). The control group was challenged with the same amount of PBS intranasal instillations on day 7-11. During excitation, the mice exhibited symptoms indicative of an asthmatic attack, including agitation, cyanosis, tachypnea, and bucking. The mice were sacrificed 72 hours after the last intranasal instillation.

BALF cells were collected by inserting a catheter into the trachea through a cervical incision and flushing the lungs with 0.7ml of ice-cold PBS. Then mice were perfused with ice-cold PBS via the right ventricle to clear blood from lung tissue. The left lung tissues were harvested for hematoxylin and eosin (HE) and periodic acid-Schiff (PAS) staining. Right lung tissues were analyzed for gene expression using quantitative reverse transcription polymerase chain reaction (qRT-PCR).

### 2.13 BALF cell counts and histological examination of lungs in mice

BALF cell pellets were stained with Wright-Giemsa staining solution (BaSO, China) and counted by two independent blinded investigators. The corresponding lung tissue sections were prepared, including pathological tissue sampling and fixation, embedding, paraffin section, and frozen section. The lung sections were stained using HE and PAS staining kit (Servicebio, China) following the provided instructions. The staining characteristics were observed with an optical microscope. HE and PAS staining were scored by a blinded observer and based on previously described methods (46). Briefly, the severity of peribronchial inflammation was evaluated on a scale from 0 to 4: 0, normal; 1, few cells; 2, a single layer of inflammatory cells 1 cell layer deep; 3, a ring 2–4 inflammatory cells deep; 4, a ring more than 4 inflammatory cells deep. The abundance of PAS-positive mucus-containing cells in each airway was scored as follows: 0 for no visible hyperplasia or mucus production, 1 for PAS-positive cells in 0–25% of bronchioles, 2 for 25–50%, 3 for 50–75%, and 4 for 75–100%.

### 2.14 Cell culture and treatments

Human normal bronchial epithelial BEAS-2B (catalog number: CRL-3588) cells from the American Type Culture Collection (ATCC) were cultured in RPMI-1640 medium (Solarbio, China) with 10% fetal bovine serum (FBS, Sigma-Aldrich, USA) and 1% penicillin/streptomycin (Solarbio, China). Cells were cultured at 37°C in a humidified atmosphere containing 5% CO_2_ at 37°C. In addition, BEAS-2B cells were passaged every 2 days following trypsin (TrypLE™ Express, gibco, Thermo Fisher Scientific, USA) digestion. Cells were seeded in a complete medium for 24h and subsequently treated with HDM (100µg/ml, Dermatophagoides Pteronyssinus, Greer Laboratories, USA) or PBS with the same amount, and the cells were harvested for qRT-PCR after 24h.

### 2.15 Quantitative reverse transcription-polymerase chain reaction

Total RNA from the right lung tissues of mice and BEAS-2B cells was extracted by AG RNAex Pro Reagent (Accurate Biology, China) according to manufacturer manual. RNA concentration was quantified and measured by absorbance at 260 nm and 280nm using a spectrophotometer (Nanodrop 2000, Thermo Fisher Scientific, USA). Then cDNA was reversely transcribed with SweScript All-in-One RT SuperMix for qPCR (One-Step gDNA Remover) (Servicebio, China) followed by real-time PCR amplification by using of SYBR Green Premix Pro Taq HS qPCR Kit (Rox Plus) (Accurate Biology, China) in QuantStudio™ 5 Real-Time PCR System (Applied Biosystems™, Thermo Fisher Scientific, USA). Finally, the RNA quantity was estimated using the 2^−ΔΔCt^ method, with β-actin as the reference gene for normalization (47). The primers employed for qRT-PCR analysis were performed in **Additional File 1**: **Supplementary Table S1**.

### 2.16 Statistical analysis

Bioinformatics statistical analysis and images were conducted using RStudio software (v2023.6.0.421). Student’s t test, Wald Chi-Squared test and Wilcoxon rank sum test were applied as specified. Correlation analysis was completed with the Spearman method. The Graphpad Prism version 10.1.2 for Windows (GraphPad Software, Boston, Massachusetts USA, www.graphpad.com) was used for qRT-PCR statistical analysis. Results were presented as mean ± standard deviation (SD). Statistical comparison was performed using the student’s t-test. *P* value < 0.05 were considered statistically significant and are represented as follows: *p<0.05; **p<0.01; ***p<0.001.

## 3 Results

### 3.1 ScRNA-seq analysis identified the diversity of epithelial cell populations in BALF

To investigate gene expression and create a detailed map of the BALF cell landscape in asthma at single-cell resolution, we analyzed the scRNA-seq data (GSE164015) from the GEO database using bioinformatics techniques (**Figure 1**). After data integration, strict quality control (QC) filtering and removing batch effect (**Additional File 2**: **Supplementary Figures S1A, B**), all BALF cells were divided into 8 clusters. The dot plot effectively displayed the expression of canonical marker genes that can distinguish epithelial cells from immune cells well (**Additional File 2**: **Supplementary Figure S1C**). Based on this, we identified 0, 2, and 4 clusters as epithelial cells and the rest clusters as immune cells (**Additional File 2**: **Supplementary Figures S1D, E**). Thus, 24,316 epithelial cells and 9,072 immune cells from 8 samples were further analyzed. The number of immune cells in A was more than C (**Additional File 2**: **Supplementary Figure S1F**). Our analysis initially focused on epithelial cells, followed by the immune compartments.

**Figure 2:**
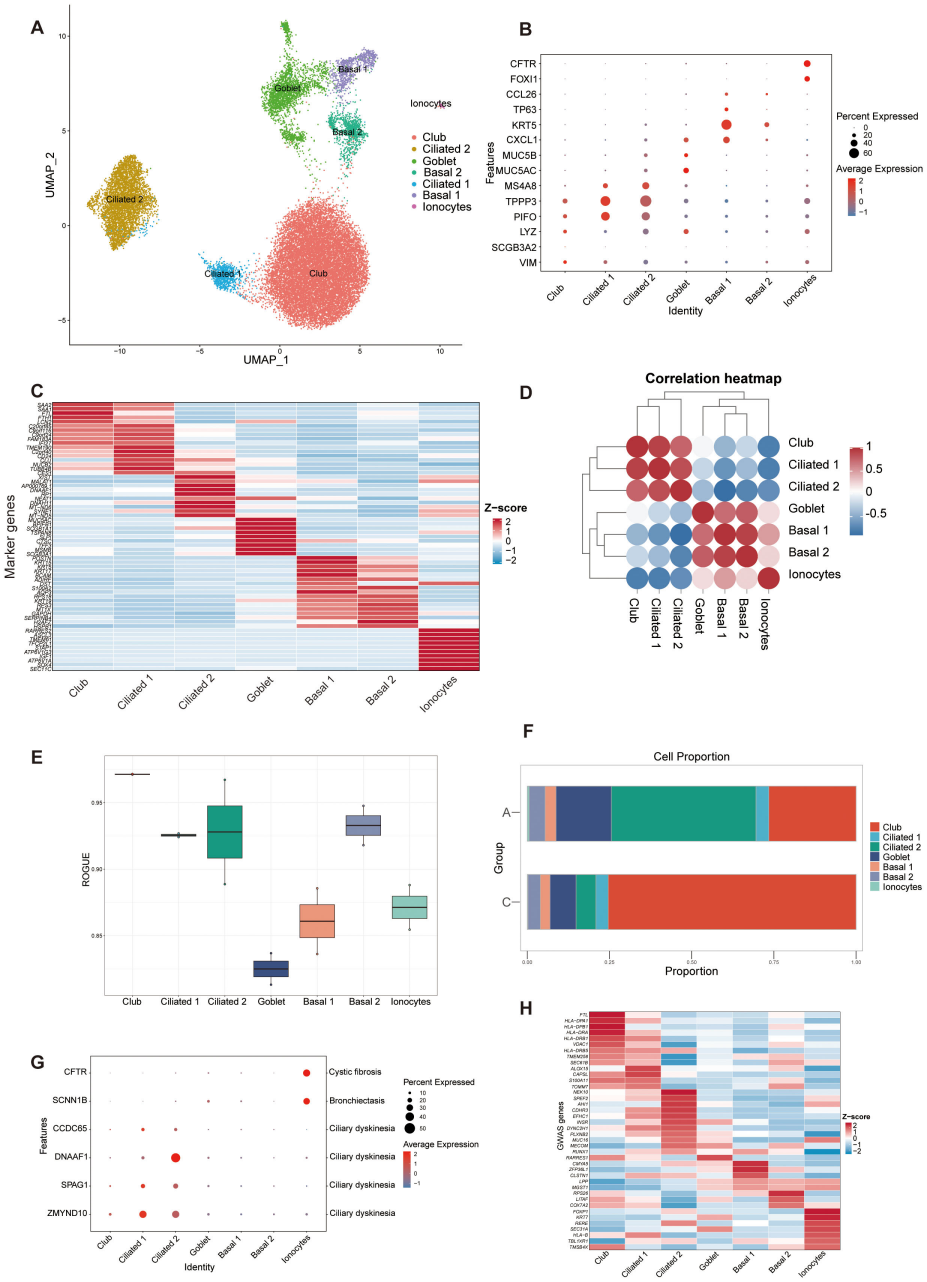
ScRNA-seq identified 7 epithelial cell clusters in BALF. **(A)** UMAP representation of 7 epithelial cell clusters from all samples. Ciliated 2, ciliated cell cluster 2; Basal 2, basal cell cluster 2; Ciliated 1, ciliated cell cluster 1; Basal 1, basal cell cluster 1. **(B)** Dot plot of canonical cell type marker genes for each cell population. **(C)** Heatmap showing the normalized average expression levels of the top differentially expressed marker genes in each cell subpopulation. **(D)** Correlation analysis between the epithelial cell populations. **(E)** Boxplot showing cell purity for each cell type by ROGUE. ROGUE, Ratio of Global Unshifted Entropy. **(F)** Bar plot of the epithelial clusters distribution in the A and C groups. **(G)** Dot plot displaying the specific gene expression levels and percentage of cells expressing genes associated with lung phenotypes. **(H)**. Heat map depicting the normalized average expression of asthma GWAS genes.

In the epithelial lineage, cells were clustered into 7 separate subsets. We identified multiple club, ciliated, goblet, basal cells, and ionocytes based on canonical markers and marker genes (**Figures 2A-C**, **Additional File 2**: **Supplementary Figure S1G**). Two distinct states were identified within both basal and ciliated epithelial cells. The two basal cell states were associated with differentiation functions. Basal 1 cells exhibited elevated expression of KRT5, TP63, and BCAM, which are involved in cell secretion and adhesion. In contrast, basal 2 cells showed higher expression of genes related to immunity and homeostasis, such as RPS18, RPS3, and MT1X (48–51). Ciliated 1 cells expressed higher levels of PIFO and TPPP3. DNAH11 and MSA48 are expressed at higher levels in ciliated 2 cells (**Figures 2B, C**) (11, 52, 53). According to Spearman’s correlation analysis, ionocytes had different transcriptional features when compared with other types of epithelial cells because they had no distinct positive correlation with other epithelial subsets (**Figure 2D**). In addition, basal 2, ciliated, and club cells demonstrated higher purity, while goblet cells exhibited greater heterogeneity (**Figure 2E**).

The proportion of epithelial subsets was different in two groups. A had more ciliated cells, while the C had more club cells (**Figure 2F**). To validate our cell type identification and investigate the gene-disease relationship, we examined the expression of genes linked to lung phenotypes in various cell types. The specific genes were selected based on Braga, F. A. V.’s study by comparing Mendelian disease-related genes from the Online Mendelian Inheritance in Man (OMIM) database to DEGs in asthma (12). The findings indicated that the epithelial lung components exhibited unique expression profiles of genes linked to Mendelian disorders, varying by cell type (**Figure 2G**). Analysis of asthma GWAS gene expression in scRNA-seq data revealed a significant role of airway epithelial cell types in asthma susceptibility with elevated expression of asthma GWAS genes in club and ciliated cells (54) (**Figure 2H**).

### 3.2 Differences in transcriptome profiles of epithelial cell subsets

**Figure 3:**
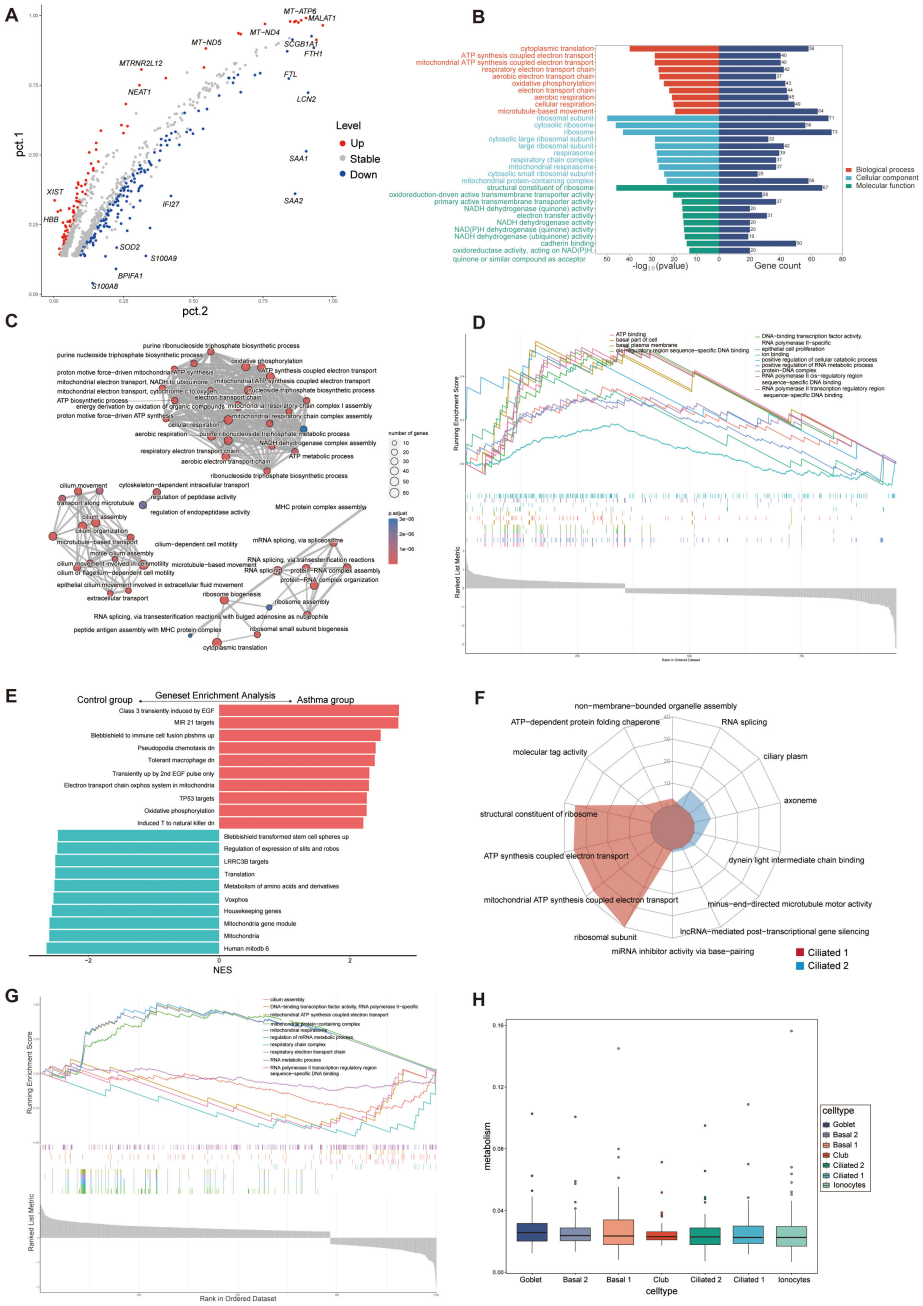
Functional enrichment and metabolism analysis of epithelial cells. **(A)** Volcano plot of DEGs for C and A groups. The top 10 upregulated genes and top 10 downregulated genes were labeled according to the value of log2FC. Pct.1 and pct.2 (normalized) indicate the proportion of the corresponding gene expression in the two groups. Stable, no significant; Up, upregulated genes; Down, downregulated genes. **(B)** GO enrichment analysis of DEGs between C and A groups. **(C)** Network diagram of GO enrichment analysis depicting the relationships between enrichment items. **(D)** GSEA of GO pathways representing some highly enriched pathways. **(E)** The GSEA of C2 pathways of the C and A groups. NES, normalized enrichment score. C2: curated gene sets in MSigDB datasets. **(F)** Radar plot showing enrichment of GO terms of ciliated 1 and ciliated 2 cells. **(G)** GSEA of GO pathways presenting some highly enriched pathways in the ciliated subsets. **(H)** Boxplot of the metabolic pathway activity in epithelial cells subsets.

According to the cut-off criteria (*p* value < 0.05 and |log2FC| > 0.5), 242 DEGs were identified between A and C, with 98 upregulated and 144 downregulated genes (**Figure 3A**). GO analysis was conducted to annotate the functions of DEGs. Pathways related to oxidative phosphorylation, respiratory electron transport chain, and ATP synthesis were significantly emphasized (**Figures 3B, C**). Single-cell GSEA analysis revealed that pathways related to transcriptional regulation, DNA and RNA metabolism, basal cellular processes, and ATP binding were upregulated in A compared to C (**Figure 3D**), confirming the association of these pathways with asthma exacerbation. It is worth noting that immune cell fusion, T cell activated, and molecule targets were upregulated in A, suggesting that these epithelial cells are crucial in connecting the immune system (**Figure 3E**).

The significant increase in ciliated 2 cells in patients experiencing acute asthma exacerbations suggests their potential role in asthma development. We compared the functional annotations of ciliated 1 and ciliated 2 cells to investigate their functions. The analysis indicated significant upregulation of several regulatory pathways, including cilium assembly, DNA-binding transcription factor activity, and RNA metabolism, in ciliated 2 cells (**Figures 3F, G**), suggesting that ciliated 1 cells were mainly involved in ATP synthesis, mitochondrial respiration of electron transport chain, and the function of ciliated 2 was mainly the movement of flagella, DNA and RNA metabolism synthesis. Goblet cells, which swiftly boost mucus production upon stimulation, also showed an increase in A. These traits reflected that exposure to allergies alters the composition of airway epithelial cells, enhances mitochondrial and ribosomal activity, activates immune signaling pathways, and reshapes the airway microenvironment.

### 3.3 Trajectory and cell-cell communication analysis revealed dynamics and molecular interactions of epithelial cell populations

To elucidate epithelial cell differentiation trajectories, a pseudotime developmental trajectory analysis was conducted, revealing potential differentiation relationships. The trajectory of epithelial cells originating from basal cell subsets in the airway wall of group A diverged into a secretory lineage, mainly comprising club cells, and a ciliated lineage, primarily consisting of ciliated cells (**Additional File 3**: **Supplementary Figures S2A, B**). In the C group, basal cells first differentiated into club cells, which then matured into goblet cells or differentiated into ciliated cells. Basal cells, the primary stem cells in the airway, possess self-renewal capabilities and can differentiate into various epithelial cell types, such as club, goblet, and ciliated cells (55). Pathway enrichment analysis revealed significant enrichment in iron ion homeostasis and ficolin-1-rich granule pathways in cells with varying differentiation fates. This suggests that iron ion regulation and ficolin-1 signaling are crucial in mediating phenotypic and functional changes during epithelial differentiation in response to different microenvironmental stimuli (**Additional File 3**: **Supplementary Figures S2C, D**). A TFs-mRNA regulatory network of the hub gene of different differentiation fates was constructed to reveal the underlying mechanism by which the hub gene regulates asthma epithelial differentiation. According to the degree of the protein-protein interaction (PPI) network, the top 10 important differential hub genes and upstream TFs were listed through the cytoHubba plugin. Combining the TFs-mRNA pairs, a TFs-mRNA regulatory network was correspondingly established, including 41 TFs and 10 hub mRNAs (**Additional File 3**: **Supplementary Figure S2E**). These findings suggested that iron metabolism and ficolin-1 signaling pathways may promote epithelial cell differentiation, drive asthma progression, and trigger exacerbation events, which has not been clarified in single-cell studies.

To characterize discrepancies in the molecular interactions between epithelial cells, we utilized CellChat to construct an extensive cell-cell communications network. A had more intercellular interaction numbers and strength than C, which was possibly due to increased interactions between basal cells and other cell groups in A group (**Additional File 3**: **Supplementary Figures S2F–H**). Basal 1 cells predominantly influenced the cell-cell communication landscape in A. The detailed ligand-receptor interactions among the 7 cell clusters were explored. Further analysis found 13 significant pathways between epithelial cell clusters in asthma exacerbation, and the most significant ligand-receptor pair was APP-CD74 (**Additional File 3**: **Supplementary Figure S2I**). We also conducted a comprehensive analysis of signaling-receptor level changes across all key pathways. Some pathways occurred active in cells of A, such as the CD99 and JAM pathways (**Additional File 3**: **Supplementary Figure S2J**). In addition, CD99 and JAM signaling only targeted basal 1 in A. Certain pathways were restricted to cells in C, such as the THBS signaling pathway that targeted basal 1 and ionocytes, and the CDH and NCAM signaling pathway that targeted ionocytes (**Additional File 3**: **Supplementary Figure S2J**). In all, the results collectively indicated that the asthma exacerbation group had its own specific signaling networks associated with the disease states in epithelial.

### 3.4 Metabolic analysis of epithelial cells

The metabolic profile of epithelial cells in asthma was elucidated by evaluating the activity scores of metabolic pathways. A comprehensive examination comparing the metabolic pathways between groups A and C revealed significant differences in 82 out of 85 pathways (**Additional File 4**: **Supplementary Figure S3A**). All cell types consistently had similar metabolic activity scores and no statistical difference between them (**Figure 3H**). An examination of pathway activities in shared cell types between A and C demonstrated a high level of concordance among the corresponding pathways (**Additional File 4**: **Supplementary Figures S3B–H**). Metabolism analysis in epithelial cells could help identify metabolite biomarkers for asthma and improve understanding of the condition’s pathophysiology.

### 3.5 Single-cell RNA-seq analysis of the immune cell composition in BALF

**Figure 4:**
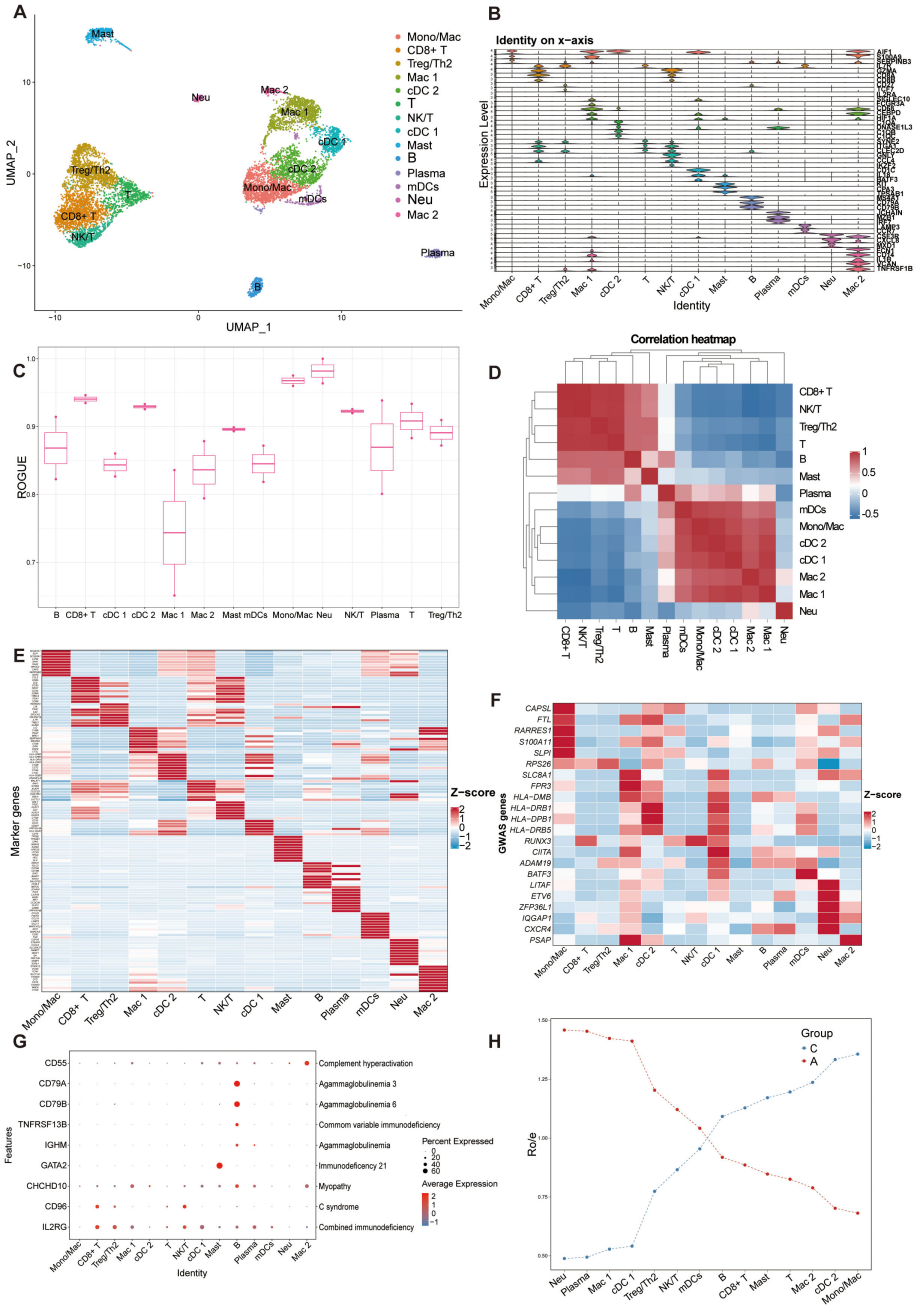
Overview of immune cell atlas in BALF with scRNA-seq detected. **(A)** UMAP representation of 14 immune cell clusters from all samples. Mono/Mac, monocytes/macrophages; CD8+ T, CD8+ T cells; Treg/Th2, Treg/Th2 cells; Mac 1, macrophages cluster 1; cDC 2, conventional dendritic cell cluster 2; T, T cells; NK/T, NK/T cells; cDC 1, conventional dendritic cell cluster 1; Mast, Mast cells; B, B cells; Plasma, plasma cells; mDCs, migratory dendritic cells; Neu, neutrophils; Mac 2, macrophages cluster 2. **(B)** Stacked violin plot of canonical cell type marker genes for each cell population. **(C)** Boxplot showing cell purity for each cell type by ROGUE. **(D)** Correlation analysis between the immune cell populations. **(E)** Heatmap showing the normalized average expression levels of the top differentially expressed marker genes in each cell subpopulation. **(F)** Heat map depicting the normalized average expression of asthma GWAS genes. **(G)** Dot plot displaying the specific gene expression levels and percentage of cells expressing genes associated with lung phenotypes. **(H)** Line chart showing lung prevalence for each cell type in C and A based on the Ro/e index.

We subsequently analyzed the single-cell transcriptomes of airway immune cells. We identified immune clusters comprising myeloid cells (macrophages, neutrophils, dendritic cells, and mast cells) and lymphoid cells (T and natural killer cells, B cells, plasma cells; **Figure 4A**, **Additional File 5**: **Supplementary Figure S4A**). The canonical markers and marker genes of these cell clusters were showed in **Figure 4B**; **Additional File 5**: **Supplementary Figure S4B**. Most cells exhibited higher purity, whereas macrophages showed higher heterogeneity (**Figure 4C**). Spearman’s correlation analysis revealed unique transcriptional characteristics in neutrophils compared to other immune cells. There was a positive correlation between myeloid cells except neutrophils, and also a positive correlation between lymphoid cells (**Figure 4D**). The expression landscapes of the top 10 feature genes in each cell subtype, as depicted in **Figure 4E**, suggested that these unique markers can accurately differentiate between cell subtypes. Comparison with GWAS analysis of human asthma genes, which showed cell-type specific expression patterns, identified multiple factors potentially influencing asthma progression (**Figure 4F**). Our study identified that conventional dendritic cell cluster 1 (cDC 1) exhibited the highest expression of asthmatic GWAS genes, with significant differential expression between the C and A groups. Additionally, the immune components displayed cell type-specific expression patterns of genes linked to Mendelian disorders, as illustrated in **Figure 4G**. Furthermore, we conducted Ro/e analysis to quantify the tissue enrichment of these populations (33). Among all populations, neutrophils, plasma cells and macrophages cluster 1 (Mac 1) were preferentially distributed in the A group, whereas Mono/Mac (monocyte/macrophage) cells, conventional dendritic cell cluster 2 (cDC 2) and macrophages cluster 2 (Mac 2) cells were preferentially located in C (**Figure 4H**).

### 3.6 Functional diversity and distinct roles of immune cell subtypes in airways

**Figure 5:**
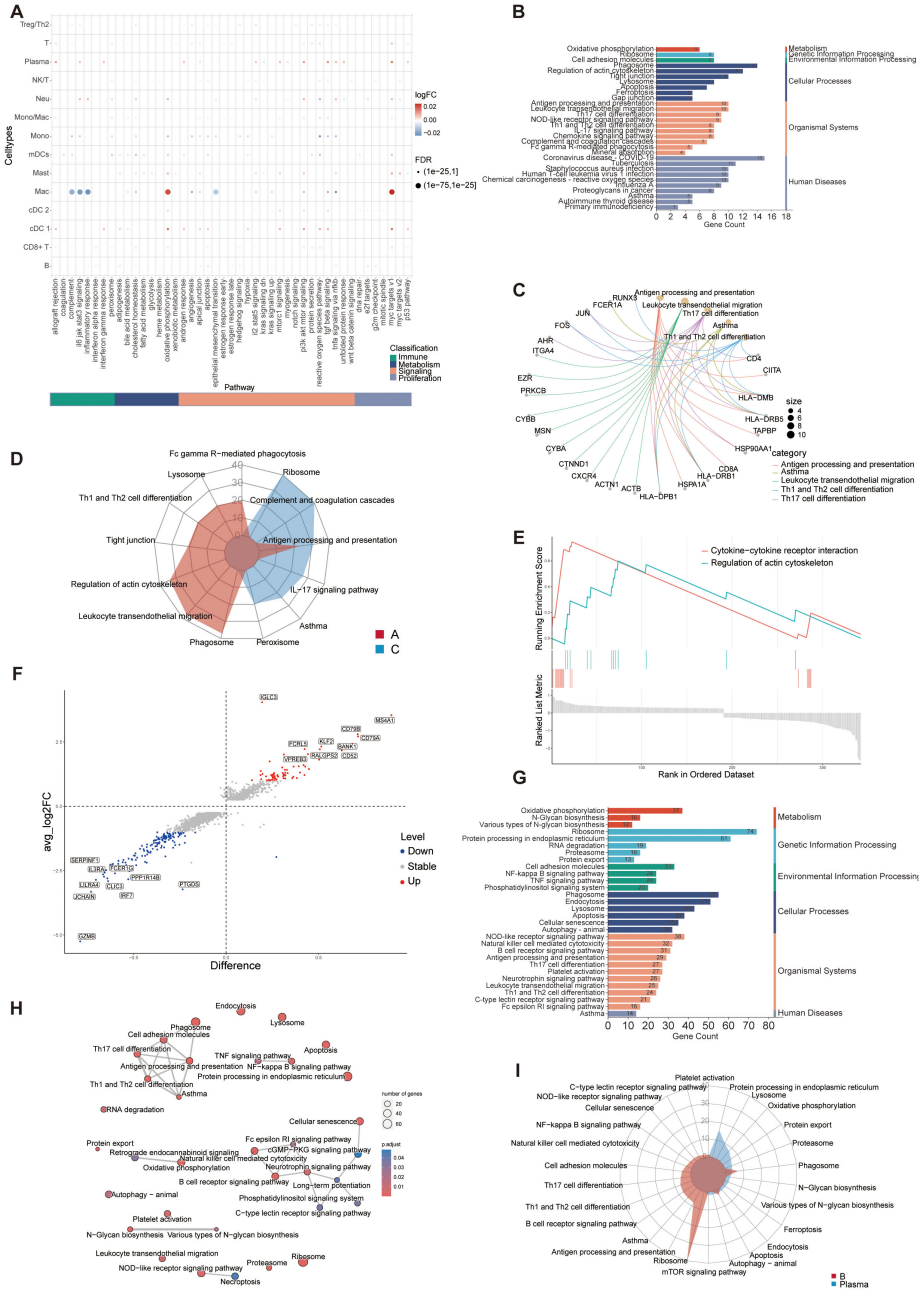
Differentially expressed immunologically relevant genes and function of immune cells in scRNA-seq dataset. **(A)** Dot plot showing differentially enriched pathways in the global immune cell type between C and A groups. **(B)** KEGG enrichment analysis of DEGs between C and **(A, C)** KEGG enrichment analysis depicting the gene regulatory network between enrichment items and related genes. **(D)** Radar plot showing enrichment of KEGG terms of C and A groups. **(E)** GSEA of KEGG pathways presenting highly enriched pathways in the A group. **(F)** Volcano plot of DEGs for C and A groups. The top 10 upregulated genes and top 10 downregulated genes were labeled according to the value of log2FC. Difference, pct.1-pct.2; Stable, no significant; Up, upregulated genes; Down, downregulated genes. **(G)** KEGG enrichment analysis of DEGs between B cells and Plasma cells. **(H)** Network diagram of KEGG enrichment analysis depicting the relationship between enrichment items. **(I)** Radar plot showing enrichment of KEGG term of B cells and Plasma cells.

To investigate discrepancies in the regulatory framework of immune cell subsets, hallmark gene sets were used to analyze pathway differences in immune cell populations between groups C and A (**Figure 5A**). Interestingly, plasma and cDC 1 cells exhibited an increase in a diverse array of pathway activities, encompassing various facets of immunology, metabolism, signaling, and proliferation. The macrophages exhibited significant up-regulation in oxidative phosphorylation and MYC targets V1, suggesting a preferential remodeling and induction of specific functional states. Furthermore, interferon (IFN)-γ response pathways were upregulated in cDC 1 cells and tumor necrosis factor (TNF)-α response was upregulated in plasma and neutrophils, with neutrophils showing greater enrichment for inflammatory response (**Figure 5A**). The KEGG functional analysis of DEGs between A and C interestingly focused on some immune-related diseases, antigen processing and presentation, Th cells signaling pathways, ferroptosis, and various signaling pathways (**Figure 5B**). The key genes associated with these immune pathways are shown in the **Figure 5C**. Genes related to leukocyte migration are relatively independent, while genes related to T cell differentiation and antigen presentation are duplicated, indicating that the differentiation and development of leukocytes in group A may have its unique regulatory mechanism. The functional radar chart can help us to see the enrichment of these pathways more intuitively in the two groups (**Figure 5D**). The A group showed notable enrichment in T cell differentiation and leukocyte migration, whereas the C was significantly enriched in ribosome and junction. While cytokine-cytokine receptor interaction and regulation of actin cytoskeleton were also upregulated in A group according to the GSEA analysis (**Figure 5E**). Taken together, these findings revealed that the complex regulation of combined innate and adaptive immune responses contributes to asthma pathogenesis.

The infiltration of plasma cells increased in A group and the number of B cells decreased in C. Further analysis was made to explore the relationship and functional differences between the two kinds of cells. The volcano plot showed the DEGs between the two types of cells (**Figure 5F**). Using KEGG molecular function terms, we found that the DEGs between B and plasma cells were highly linked to various immune-related signaling pathways, such as NOD-like receptors, NF-κB signaling, TNF signaling, and cGMP-PKG signaling pathways, suggesting that these pathways may facilitate plasma cells enrichment in asthmatic airways (**Figure 5G**). The correlation between these functions was showed in **Figure 5H**. The elevated expression of genes associated with signaling pathways and the enrichment of responses observed in this study in response to inflammation or allergens may significantly impact the composition and function of B and plasma cells. The aforementioned findings indicated that the immune microenvironment plays a significant role in shaping the functional and phenotypic diversity observed within the plasma cell and B cell repertoire. B cells terminally differentiate into plasma cells, which play significant roles in the biology of antigen specific antibody secretion (56). B cells showed specific enrichment in signaling pathways associated with differentiation, whereas the plasma cells were significantly enriched in biological processes related to protein production and export (**Figure 5I**).

Dendritic cells (DCs), as potent antigen-presenting cells, are crucial in the pathophysiology of asthma (57). To investigate immunological changes in asthma, we analyzed the single-cell transcriptomes of airway lung DCs. We identified 3 DC subsets, cDC 1 specifically expressed BATF3, KLF4, and CD1C, primarily associated with DNA-binding transcription and antigen presentation (**Additional File 6**: **Supplementary Figure S5A**). cDC 2 specifically expressed genes which are produced by immature dendritic cells involved in the classical complement pathway (e.g., C1QA, C1QB, C1QC). cDC 2 also largely expressed genes related to presenting peptides derived from extracellular proteins (e.g., HLA-DPB1, HLA-DRA, HLA-DQB1) (**Figure 4E**). mDCs specifically expressed CCR7, CCL19, and FSCN1, genes primarily associated with immune cell migration, motility, adhesion, and cellular interactions (**Additional File 6**: **Supplementary Figure S5A**) (58–60). The proportion of each subtype in A differed from that in C (**Figure 4H**). The DEGs were compared between DCs and other cell types by volcano plot (**Additional File 6**: **Supplementary Figure S5B**). The DEGs in the GO terms and KEGG pathways were closely linked to initiating and regulating immune responses, supported by the superior antigen presentation ability of DCs (**Additional File 6**: **Supplementary Figure S5C, D**). Besides, GSEA analysis confirmed our findings by revealing a strong enrichment for immune-related pathways. It is worth noting that dendritic cell maturation, IFN-γ signaling, antigen processing and presentation were upregulated in DCs, highlighting the crucial role of IFN-γ signaling in the immune response of DCs (**Additional File 6**: **Supplementary Figure S5E**).

### 3.7 Analysis of differentiation of macrophages in BALF

**Figure 6:**
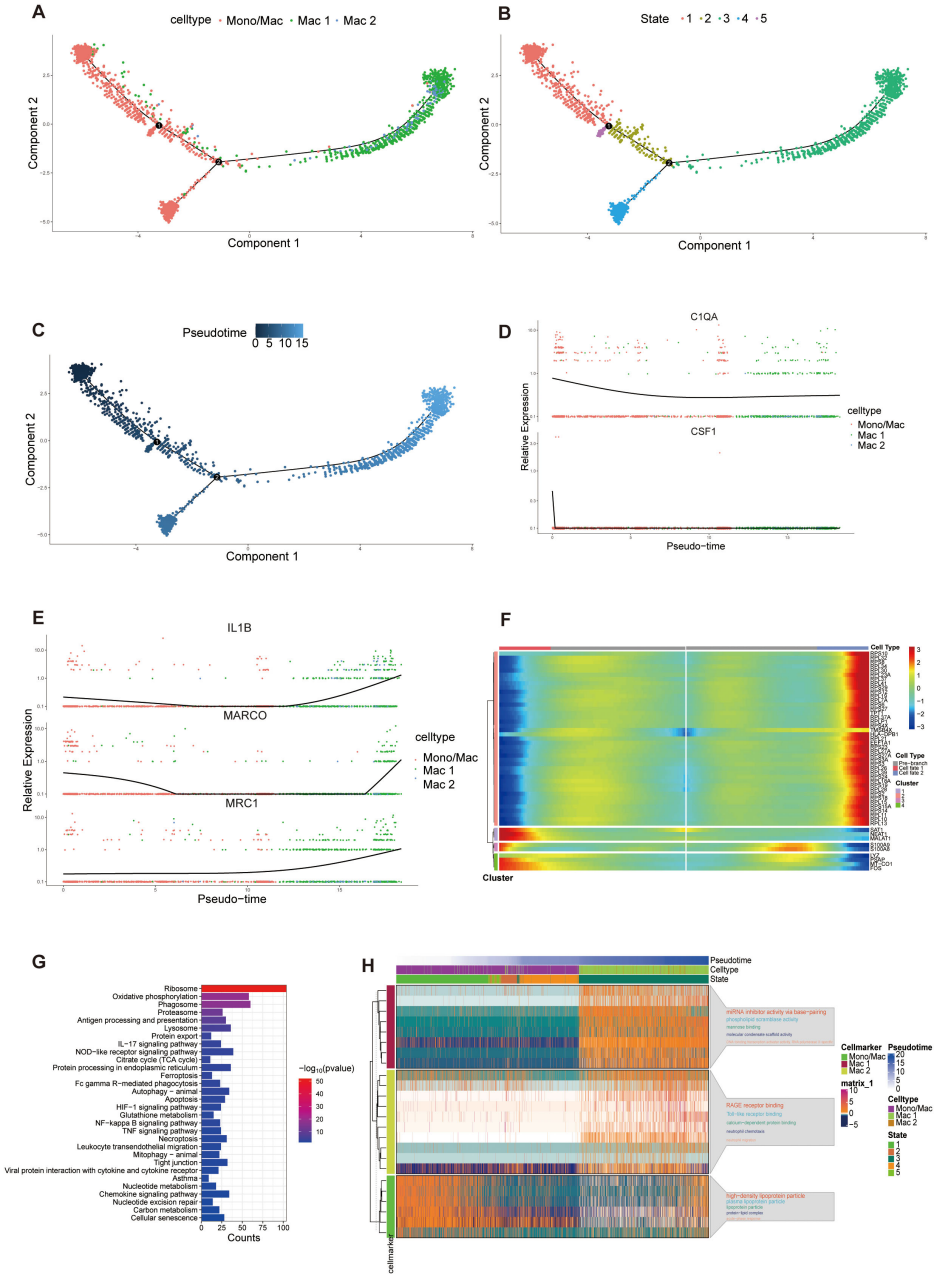
Pseudotime analysis of macrophage populations. **(A-C)** Trajectory analysis reveals the differentiation process of macrophage populations, colored-coded by the associated cell subpopulations **(A)**, states **(B)** and pseudotime **(C)**. Trajectory directions were determined by a comprehensive consideration of biological prior and pseudotime analysis. **(D, E)** Scatter plots showing the expression of selected genes in the pseudotime progresses. **(F)** Heatmap displaying the dynamic expression of fate-determining genes which were obtained by BEAM analysis along the pseudotime trajectory, and these genes were clustered into 4 groups according to their expression pattern along the pseudotime. **(G)** KEGG enrichment analysis of differential related genes in heatmap. **(H)** Heatmap of the expression of gene markers in each macrophage cluster along the pseudotime trajectory (left) and selected GO pathways related to corresponding gene markers. The top 5 GO pathways were displayed according to *p* value (right). The smaller the *p* value, the larger the font of the pathway name.

Macrophages are a heterogeneous and dynamic population of cells, which can differentiate from monocytes or develop from the proliferation of resident macrophages (61). ScRNA-seq analysis identified three populations, all exhibiting high AIF1 expression (**Figure 4B**). Mac 1 exhibited elevated SIGLEC10 and FCGR3A expression, with comparatively lower CEBPD levels. Mac 2 exhibited high FCN1 expression and several monocytes marker genes, including CD14, IL1B and VCAN (62), while showing low to no expression of SIGLEC10 and FCGR3A (63). The third population of cells had common biomarkers with monocytes and macrophages which did not distinguish this subset well, therefore we clustered it to monocyte/macrophage. To further understand the immune dynamics, we used the Monocle analysis toolkit to perform cell trajectory analysis to explore the potential transitions between cell types. According to the trajectory analysis, we found that monocyte/macrophage cells can transform into the two macrophage clusters over time which may have significantly different biological functions. This trajectory can be segmented into 5 distinct states (**Figures 6A–C**). C1QA and CSF1 are the marker genes of tissue-resident macrophages and their expression all decreased significantly along the pseudotime (64, 65) (**Figure 6D**). On the other hand, the expression of MRC1 (the marker gene of M2 macrophages), along with MARCO and IL1B (the marker genes of M1 macrophages) increased over time in cluster 2, suggesting a mixed population of M1 and M2 macrophages (**Figure 6E**) (66). Based on biomarkers expression and pseudotime analysis, we inferred that Mac 1, which increased significantly in A group, may be derived from tissue-resident macrophages, while Mac 2 from differentiated monocytes. We analyzed the cell trajectory from left to right and categorized the genes into two clusters. BEAM analysis displayed the fate-determining genes related to the differentiation of pre-branch to cell fate 1 and cell fate 2 (**Figure 6F**). KEGG analysis of these branch-related differential genes showed that the IL17, NOD-like receptor, HIF-1, and NF-κB signaling pathway mediated the phenotypic and functional shift during macrophage differentiation, supporting the possibility that these signaling pathways play important roles in the progression of asthma (**Figure 6G**). GO analysis of cell marker genes from three macrophage clusters identified 15 signaling pathways. Among them, metabolic pathways and molecular signaling pathways were closely associated with asthma formation (**Figure 6H**). Collectively, these results indicated that tissue-resident macrophages in the A group undergo a differentiation process into Mac 1, which is significantly enhanced. Multiple signaling pathways were identified as playing a role in promoting this differentiation process.

### 3.8 Intercellular communications of immune cells

**Figure 7:**
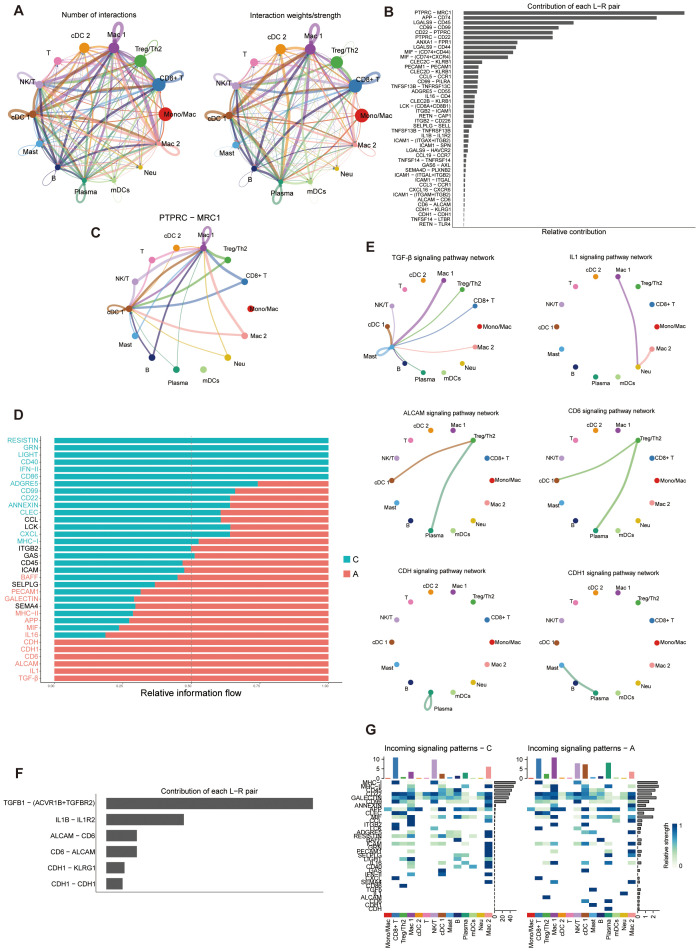
Cell-cell communications of immune cell populations. **(A)** Illustration of the Interaction numbers (left) and interaction weights/strength (right) between immune cell types. **(B)** Relative contribution of each ligand-receptor pair to the overall communication network. L-R, Ligand-receptor. **(C)** The PTPRC-MRC1 pair interactions network between immune cell types. **(D)** Significant signaling pathways ranked based on differences in the overall information flow within the inferred networks between C and A groups. Pathways enriched in A group are red; black pathways are equally enriched in A and C groups; green pathways are enriched in C group. **(E)** Inferred signaling networks enriched in **(A, F)** Relative contribution of each ligand-receptor pair to the overall communication network of inferred signaling networks which enriched in **(A, G)** Analysis of the interactions between C and A groups on the activity of incoming signaling pathways.

Extensive cell-cell communications were demonstrated among the immune cell clusters using Cellchat. The number and strength of cell-cell communication among immune cells were elevated (**Figure 7A**). The PTPRC-MRC1 ligand-receptor pair was the most significant, involving the majority of immune cell clusters (**Figures 7B, C**). Through a comparative analysis of the information flow in the C and A groups, we discovered 34 signaling pathways that exhibited enrichment in either group, with additional pathways showing equal enrichment in both regions (**Figure 7D**). Of particular interest, certain pathways enriched in the A group have been linked to the development of asthma. Previous data indicated an upregulation of TGF-β in asthma (67). TGF-β is crucial in regulating cellular processes such as epithelial cell growth suppression, epithelial cell differentiation, fibroblast activation, and extracellular matrix organization (68). TGF-β also plays a crucial role in T cell differentiation and is significantly involved in asthmatic airway inflammation (69). Pathways that enriched in the A group were showed in **Figure 7E**. At the single-cell level, we demonstrated that TGF-β dependent signaling was transmitted from certain immune cells to mast cells, primarily involving TGFβ-(ACVR1B+TGFBR2) interactions among all known ligand-receptor pairs (**Figure 7F**). Subsequently, a detailed analysis of signaling-receptor level changes across all significant pathways was conducted. The study identified active pathways in group A, including the IL1 pathway targeting neutrophils (**Figure 7G**). ALCAM signaling only targeted Treg/Th2 cells in A group. Certain pathways were restricted to cells in C, such as the CD86 signaling pathway that targeted Treg/Th2 cells, and the GRN signaling pathway that targeted monocytes. Some of the remaining pathways exhibited variations corresponding to disease states. For example, SEMA4 signaling mainly targeted macrophages and monocytes in the A group, whereas it only targeted monocytes in the C group (**Figure 7G**). In addition, CCL signaling targeted monocytes and mDCs in A group, whereas it targeted macrophages and monocytes in C group (**Figure 7G**). These characteristics may reflect that the changed interactions and communications of immune cells reshape the asthma microenvironment.

### 3.9 Observation of a cell−type−specific metabolic program

Immunometabolism and the associated phenotypic biology in asthma remain unclear (70). To comprehend the metabolic profile of immune cells in asthma, the metabolic activity scores of all 85 active metabolic pathways were computed. Among all cell types, macrophage cells demonstrated consistently elevated metabolic activity scores in both C and A groups (**Additional File 7**: **Supplementary Figures S6A, B**). Further analysis of the metabolic pathways differences between the two groups identified 28 potentially upregulated pathways in the A group, indicating strong metabolic profiles associated with asthma (**Additional File 7**: **Supplementary Figure S6C**). Notably, some upregulated metabolic pathways in the A group are implicated in asthma pathogenesis. For example, monocytes derived from asthmatic patients, as well as lung tissues from ovalbumin-sensitized and challenged mice, exhibited elevated levels of lactate and enhanced aerobic glycolysis (71). Moreover, glutathione-S-transferase P (GSTP) induces aerobic glycolysis in bronchial epithelial cells, highlighting the importance of the glutathione–glycolysis signature in asthma pathogenesis (72). In A group, glutathione, purine, glycolysis, and oxidative phosphorylation which are involved in mitochondrial redox system significantly up-regulated, suggesting that A group may require more ATP production (**Additional File 7**: **Supplementary Figure S6D**). These findings revealed metabolic pathways in different immune cells that could lead to immune response dysregulation in asthma. New therapies that target the critical biological mediators in the metabolic pathways in asthma can be explored further.

### 3.10 Integrating single-cell and bulk transcriptome analysis and key regulated gene identified

**Figure 8:**
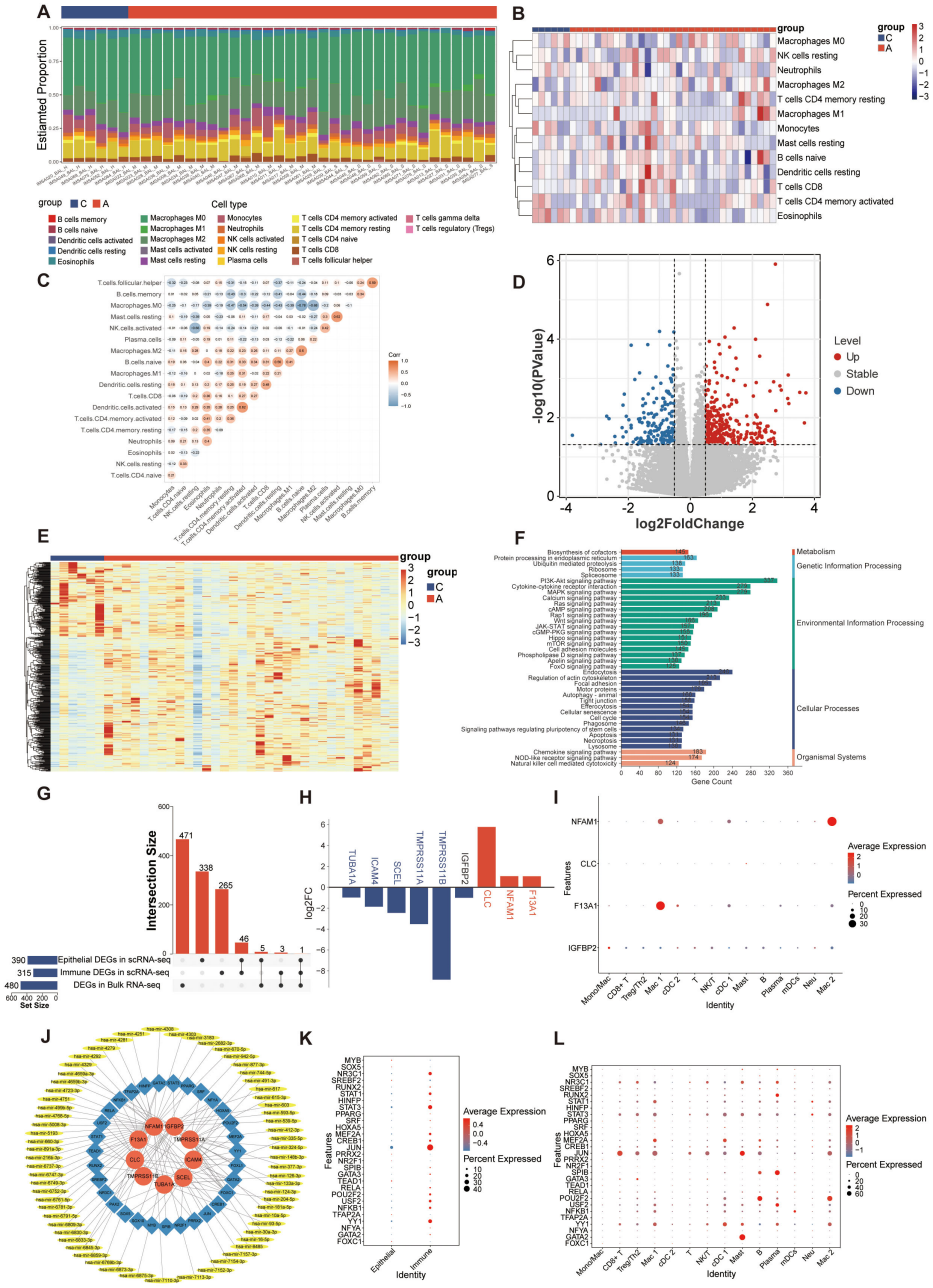
Analysis of bulk RNA-seq dataset and potential regulatory mechanisms. **(A)** Immune infiltration proportion of the 22 immune cell populations in 39 samples. **(B)** Heatmap exhibiting the expression landscapes of infiltrated immune cells between C and A groups. **(C)** Correlation heatmap depicting co-expression patterns among immune cells. **(D)** Volcano plot of DEGs between C and A groups in the bulk RNA-seq dataset (|log2FC|> 0.5 and *p* value < 0.05). **(E)** Heatmap of DEGs with unsupervised clustering in the bulk RNA-seq dataset. **(F)** KEGG enrichment analysis of DEGs between C and A groups. **(G)** Upset plot of key regulatory genes identified in scRNA-seq and bulk RNA-seq. **(H)** Expression of key regulatory genes in scRNA-seq data set. Up-regulated genes were colored in the red bar, and down-regulated genes were colored in the blue bar. The blue gene names represent the DEGs in the epithelial cells, the red gene names represent the DEGs in the immune cells and the black gene name represents the DEG in both epithelial cells and immune cells. **(I)** Dot plot showing the expression of key regulatory genes in immune cells from the scRNA-seq data set. **(J)** The TF-mRNA-miRNA regulatory network visualized by Cytoscape. Red represented mRNAs, blue represented TFs and yellow represented miRNAs. **(K)** Dot plot showing the expression of predicted TFs in scRNA-seq data set. **(L)** Dot plot showing the expression of predicted TFs in immune cells from the scRNA-seq data set.

Given the significant impact of alterations in the immune microenvironment on asthma, we conducted an analysis of the proportions of 22 immune cell types in bulk RNA-seq samples using the CIBERSORT algorithm (**Figure 8A**). Removing the cell types that expressed zero in more than half of the samples, the heatmap illustrating immune cell abundance per sample is presented in **Figure 8B**. Furthermore, the correlation among immune cells in these samples was analyzed (**Figure 8C**). A positive correlation was found between mast and activated NK cells, activated dendritic cells and CD4 memory T cells (r=0.62). Conversely, macrophages exhibited the strongest negative correlation with naïve B cells (r=-0.78). Later, we analyzed the DEGs in the bulk transcriptomic data. A total of 480 dysregulated DEGs were retained between A and C groups in GSE136587 (**Figure 8D**). A heatmap of the upregulated and downregulated DEGs demonstrated relative consistency within groups (**Figure 8E**). The functional analysis of DEGs in asthma interestingly highlighted various signaling pathways and immune responses (**Figure 8F**), consistent with the scRNA-seq analysis of GSE164015.

To identify key regulatory genes involved in both acute asthma exacerbation and its pathogenesis, we analyzed the common expression patterns of DEGs across two datasets. As epithelial and immune cells showed different characteristics in asthma, the key regulatory genes were obtained by intersecting of the DEGs with the most significant difference (*p* value <0.05 and |log2FC| > 0.5) between C and A group in epithelial, immune cells and bulk RNA-seq. The change trend of DEGs in each dataset should be in the same direction. 9 key regulatory genes crucial to asthma development were identified (**Figure 8G**). Among them, 5 genes (*tmprss11a*, *tuba1a*, *scel*, *icam4*, and *tmprss11b*) down-regulated were in airway epithelial cells, 3 genes (*clc*, *nfam1*, and *f13a1*) were highly expressed in immune cells, and 1 gene (*igfbp2*) down-regulated played critical roles in both epithelial and immune cells in scRNA-seq data (**Figure 8H**). NFAM1, F13A1, and IGFBP2 were mainly expressed in macrophages which at different stages of differentiation. CLC was mainly expressed by mast cells (**Figure 8I**). However, the 9 key regulated genes did not show a tendency of change by the moderate and severe degree of asthma in bulk RNA-seq (data were not showed).

To better understand how key regulated genes influence asthma progression, we explored upstream regulation by predicting related TFs and miRNAs. The TFs-mRNA-miRNA regulatory network, constructed using NetworkAnalyst, includes 105 nodes and 119 edges (41). A total of 31 TFs genes and 65 miRNAs interacted with the 9 key regulated genes (**Figure 8J**). A total of 28 transcription factors were detected in scRNA-seq data, of which MYB, SOX5, SREBF2, PRRX2, NR2F1, TEAD1, TFAP2A, and FOXC1 were mainly expressed in epithelial cells and the rest were mainly expressed in immune cells (**Figure 8K**). These TFs were expressed in one or more subtypes of immune subtypes, with all immune cells showing activation in TFs expression (**Figure 8L**).

### 3.11 Experiments validations of key regulated genes expression in asthma

**Figure 9:**
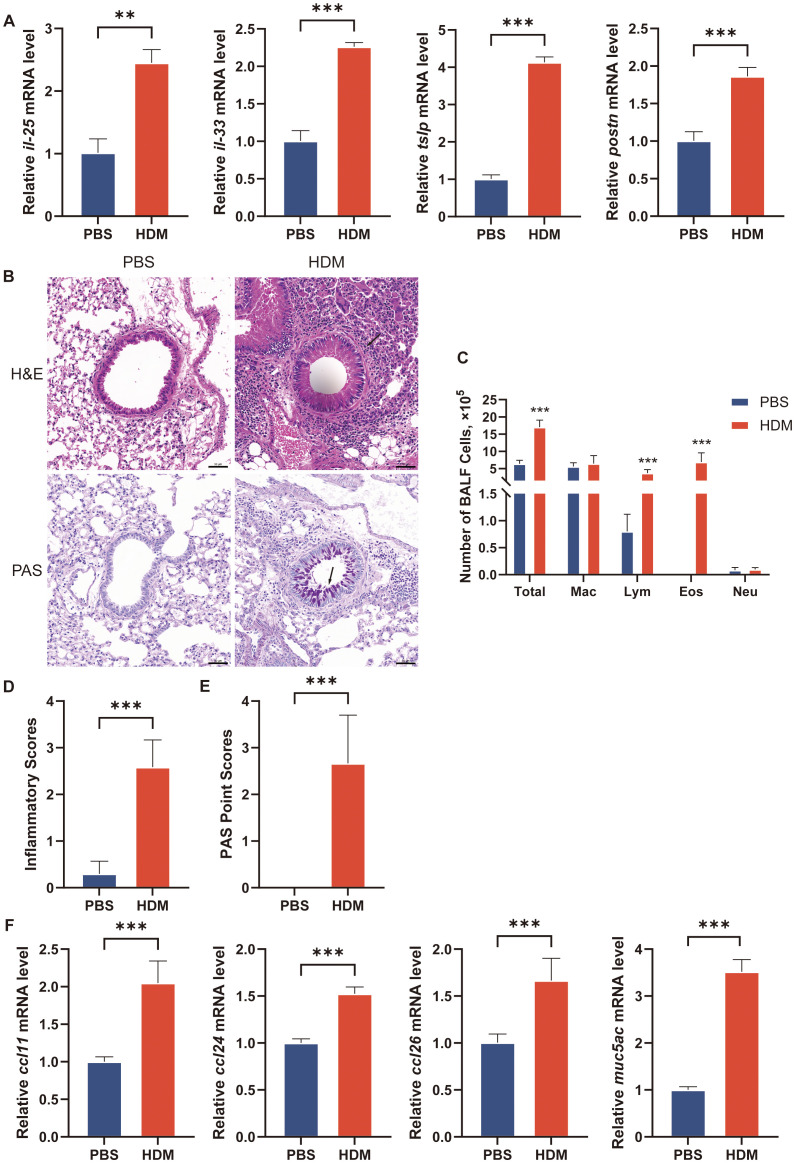
Establishment of asthma model in cell line and mouse. **(A)** The transcriptional levels of *il-25*, *il-33*, *tslp* and *postn* in culture medium of BEAS-2B cells after treatment with PBS or HDM for 24h. **(B)** H&E staining and PAS staining of representative lung sections. Black arrowheads indicate inflammatory infiltration after H&E staining and goblet cells containing mucus (magenta) after PAS staining. **(C)** Counts for macrophages, eosinophils, lymphocytes and neutrophils in BALF. n = 6 mice per group. BALF, bronchoalveolar lavage fluid. Total, Total cells number in BALF; Mac, macrophages; Lym, lymphocytes; Eos, eosinophils; Neu, neutrophils. **(D)** Inflammatory scores of lung sections from mice intranasally challenged with HDM or PBS were calculated as described in Materials and methods. **(E)** PAS point scores of lung sections from mice intranasally challenged with HDM or PBS were calculated as described in Materials and methods. **(F)** The transcriptional levels of *ccl11*, *ccl24*, *ccl26* and *muc5ac* in lung of mice. The data are represented as mean ± SD. **p<0.01; ***p<0.001.

**Figure 10:**
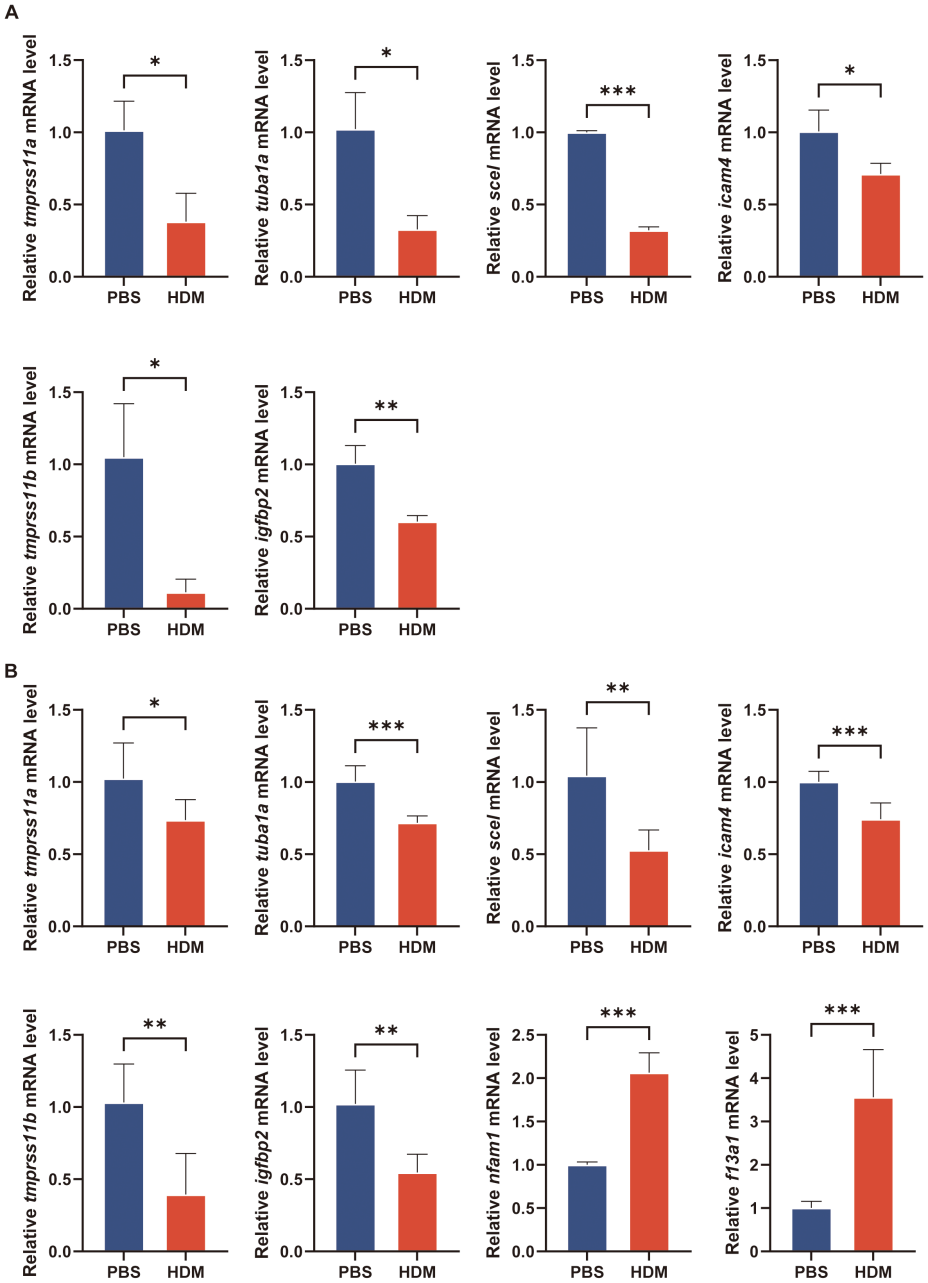
Verification of key regulatory genes in asthma model by qRT-PCR. **(A)** The transcriptional levels of *tmprss11a*, *tuba1a*, *scel*, *icam4*, *tmprss11b* and *igfbp2* in culture medium of BEAS-2B cells after treatment with PBS or HDM for 24h. **(B)** The transcriptional levels of *tmprss11a*, *tuba1a*, *scel*, *icam4*, *tmprss11b*, *igfbp2, nfam1* and *f13a1* in lung of mice. The data are represented as mean ± SD. *p<0.05; **p<0.01; ***p<0.001.

We next aimed to preliminary verify our findings that these key regulated genes are involved in asthma by *in vitro* and *in vivo* experiments. We established a cellular model of asthma by stimulating BEAS-2B cells with HDM. The inflammatory cytokines (*il-25*, *il-33*, *tslp*, and *postn*) mRNA increased in HDM-stimulated BEAS-2B cells (**Figure 9A**). We quantified the mRNA levels of key epithelial cell genes using qRT-PCR. *Tmprss11a*, *tuba1a*, *scel*, *icam4*, *tmprss11b*, and *igfbp2* mRNA expression was significantly decreased in the HDM group compared to PBS group (**Figure 10A**).

The mice were intranasally administered HDM or PBS for sensitization and challenge. HDM-challenged mice exhibited significantly higher total cell and eosinophil counts in BALF, as well as increased inflammatory cell numbers around the conducting airways, as assessed by H&E staining (**Figures 9B–D**). PAS staining revealed numerous mucus-containing epithelial cells, and *muc5ac* transcript levels were significantly elevated in HDM-challenged mice compared to PBS-challenged mice (**Figures 9E–F**). We analyzed the expression of the eotaxins (*ccl11*, *ccl24*, and *ccl26*) in mouse lungs by qRT-PCR. HDM challenge induced the mRNA expression of *ccl11*, *ccl24*, and *ccl26* in lung tissue of mice (**Figure 9F**). Due to species difference between human and mouse, the gene information for CLC in mice was not available in NCBI, so there was no *in vivo* experimental verification of CLC. The *nfam1* and *f13a1* mRNA expression was significantly increased and *tmprss11a*, *tuba1a*, *scel*, *icam4*, *tmprss11b*, and *igfbp2* expression was decreased in HDM-challenged mice (**Figure 10B**). Taken together, our data indicated that TMPRSS11A, TUBA1A, SCEL, ICAM4, TMPPRSS11B, IGFBP2, CLC, NFAM1 and F13A1 are crucial in the occurrence and development of asthma.

## 4 Discussion

Airway BALF are important for providing further insight into altered epithelial cells and immunity pathways of asthma patients. “Omic” techniques on them help to clarify the pathophysiology of delayed reaction induced by allergen in asthma more precisely (73). This study directly compared exacerbated allergic asthmatics with allergic asthmatic controls to identify distinct populations, functional properties, and tissue-specific trajectories of epithelial and immune cell subsets in BALF at the single-cell level. Furthermore, a single-cell resolution landscape of cellular metabolisms and communication networks was constructed. The hub conclusions were displayed in **Table 1**. Then we explored 9 key regulatory genes associated with asthma occurrence and development by performing a series of bioinformatics analyses based on scRNA-seq and bulk RNA-seq. TFs-mRNA-miRNA networks associated with the nine key regulated genes were constructed. Afterward, we preliminary verified these key regulatory genes in epithelial cell and mouse asthma models to offer new insight into the pathogenesis of allergic asthma.

**Table 1:** The characteristics of epithelial and immune cells in asthma excerbation.

Celltypes	Analysis	Hub conclusions	Fig
Epithelial	Functional Enrichment	The number of goblet and ciliated cells increased significantly, mitochondrial and ribosomal activity enhanced.	**Figures 2**, **3**
	Pseudotime	Iron metabolism and ficolin-1 signal related pathways promote epithelial cell differentiation.	**Supplementary Figure S2**
	Communication	APP–CD74 was the most significant ligand–receptor pair. CD99 and JAM pathways were occurred active.	**Supplementary Figure S2**
	Metabolism	Epithelial cell populations had the similar metabolic activity scores. 47 metabolic pathways were upregulated.	**Supplementary Figure S3**
Immune	Functional Enrichment	A complex regulation of conjoint innate and adaptive immune responses activated.	**Figure 5**
	Pseudotime	Tissue-resident macrophages undergo a differentiation process into Mac 1, which is significantly enhanced. IL17, NOD-like receptor, HIF-1, and NF-κB signaling pathway mediated the phenotypic and functional shift.	**Figure 6**
	Communication	TGF-β dependent signaling was upregulated significantly.	**Figure 7**
	Metabolism	Macrophages cells had the highest metabolic activity scores. 28 metabolic pathways were upregulated.	**Supplementary Figure S6**

Mucociliary clearance dysfunction has been reported in asthma in the stable state and during exacerbations (74). Analysis of the scRNA-seq epithelial data revealed that the number of ciliated cells increased significantly and respiratory electron transport chain, ATP synthesis and NAD(P)H dehydrogenase (quinone) activity were primarily concentrated on in A group. These results confirm that respiratory ciliated cells require an efficient ATP supply chain for cilia beating to clear mucus; however, this organization also produces reactive oxygen species (ROS), which risk causing injury (75). Pseudotime analysis enhances our understanding of epithelial cell changes and dynamic gene regulatory programs throughout continuous biological processes (76). Results indicated that basal cells act as progenitors, differentiating into ciliated, secretory, and goblet cells during repair. The secretory and ciliated lineages in asthma also underscore the essential role of ciliated cell function and mucociliary clearance in respiratory tract defense. Besides, GO results prompted that epithelial cells differentiation are related to iron homeostasis and transport. The findings elucidated the regulatory impact of iron overload on epithelial cell proliferation and differentiation, offering new insights into the mechanisms by which iron overload influences asthma. Ferroptosis was recognized as a non-apoptotic cell death type linked to asthma (77). Our study further identified a potential possibility that iron overload promotes the abnormal differentiation of epithelial cells. Fcolin-1-rich granule is also one of the important mechanisms affecting epithelial cell differentiation. Gao Pengfei et al. reported that asthmatic patients exhibited elevated plasma ficolin-1 concentrations, which diminished following inhaled corticosteroids (ICS) treatment, suggesting ficolin-1’s potential role in asthma pathogenesis (78). Cell-cell communication analysis indicated that signal-communication patterns were altered in asthma exacerbation. It was noticed that the MDK–NCL ligand-receptor pair showed higher activity and possibility between epithelial cells in A group, indicating that NCL might be a significant receptor during cellular cross-talk in asthma exacerbation. Furthermore, allergic exposure can alter the metabolic biology of airway epithelial cells. Metabolic analysis revealed that the metabolic activities of A and C groups were similar, but unique metabolic heterogeneity in asthma exacerbation was displayed, such as enhanced activity of glycolysis, oxidative phosphorylation pathways, purine metabolism, and unsaturated fatty acids biosynthesis pathways. A prior study utilized lipidome and metabolome analyses showed the metabolic differences in bronchial epithelial cells between asthma patients and healthy individuals. These differences are associated with inflammation and asthma severity (79).

The relationship between the immune responses of different immune subtypes is very complex. In asthma, memory IgG-positive B cells produce IgE upon stimulation and differentiate into long-lived plasma cells, which are an independent negative prognostic factor, possibly due to their production of the immunosuppressive cytokine IL-35 (80) (81). B cells also contribute to allergic inflammation through interactions with T cells (82). DCs have emerged as critical players in the communication between the innate and adaptive immune systems during the initiation and maintenance of asthma (83). In our functional enrichment analysis of DCs, results showed a significant increase in IFN-γ which induces the generation and maturation of immune-regulatory DCs (84). Moreover, IFN-γ directs DC-mediated polarization of T cells toward Th1, essential for initiating effective immune responses (85).

Macrophages, the primary immune cells in the lung, play a crucial role in the development of airway inflammation caused by environmental allergens in asthma (86). Lung macrophages exhibit heterogeneity due to their potential origins from either the differentiation of bone marrow-derived monocytes or from the proliferation of resident macrophages (87). Our pseudotime analysis of macrophages also clarified the possible source and type in the BALF of asthma exacerbation. It is crucial to acknowledge that macrophages exhibit a spectrum of polarization states, adopting intermediary phenotypes and diverse subpopulations to perform various physiological functions (61). The co-expression of gene markers for both M1 and M2 phenotypes in individual cells highlights the complexity of polarization states, complicating the assessment of macrophage generation and maturation. M1 macrophages, known for their pro-inflammatory phenotype, express cytokines and chemokines to recruit immune cells and exhibit strong phagocytic and cytotoxic abilities (88). M2 macrophages exhibit greater functional diversity including clearance of dead cells and anti-inflammatory responses, manifesting as distinct subtypes (M2a, M2b, M2c, M2d) characterized by unique profiles of cytokines, chemokines, and growth factors (89). Increased expression of MARCO and IL1B indicates promoted M1 polarization, while elevated MRC1 expression signifies enhanced M2 polarization. Given the plasticity and complexity of lung macrophage phenotypes, further comprehensive studies on macrophage polarization in both stable and exacerbating asthma are necessary.

As expected, the A group exhibited a notable increase in both the number and strength of cell communication, along with a significant enhancement of the TGF-β signaling pathway. In the functional enrichment analysis using hallmark gene sets, the TGF-β signaling pathway was also enriched and enhanced, indicating the key role of TGF-β signaling pathway in asthma exacerbation. According to inferred signaling network plots, the recipients of TGF-β signaling were closely related to mast cells in asthma. TGF-β promotes inflammation by inducing Th17 cell differentiation through Smad and p38^MAPK^ pathways and serves as a chemoattractant for monocytes, mast cells, and granulocytes (90). The ubiquitous presence of TGF-β receptors across various immune cell types suggests a significant impact on immune responses, posing a challenge in the investigation of TGF-β therapy. Furthermore, metabolic reprogramming is essential for inducing immune responses, as immune cells engage in cellular immune signaling and significantly alter metabolic pathways, including glutathione, purine, glycolysis, oxidative phosphorylation, and fatty acid metabolism, thereby enhancing their ability to respond to subsequent stimuli (91).

In addition, our study identified 9 key regulatory genes with significantly different expression between groups A and C based on combined analysis. The down-regulated genes in epithelial associated with barrier function, cell motility, adhesion, secretion, and regulation. TUBA1A encoding tubulin proteins, together with microtubule-associated proteins (MAPs) and motor proteins on the outer surface, are involved in significant cellular activities such as intracellular transport, cell division, and migration (92). Research revealed that the expression levels of TUBA1A were lower in cells exposed to particulate matter (PM10) for 24 hours than in the control group (93). This may indicate that the microtubule aggregation dynamics in asthma are disrupted. As a member of the adhesion molecular protein family, ICAM4 is not only responsible for cell adhesion, but also important for cellular, proliferation, inflammation and immune responses (94, 95). Sciellin (SCEL), a precursor to the cornified envelope of human keratinocytes, play a major role in the physical barrier properties of the stratum corneum (96). Mice with knockout of the gene encoding epidermal protein have a defect in barrier function and respond abnormally to the application of irritants (97, 98). The airway epithelial type II transmembrane serine proteases, TMPRSS11A and TMPRSS11B, are linked to SARS, MERS, and COVID-19 infections. They assist in the fusion of the virus with the cell membrane, cleave the viral spike protein, and play a regulatory role in airway epithelial cells (99, 100). The secreted protein IGFBP2 governs the distribution, function, and activity of insulin-like growth factor (IGF) in the extracellular matrix, enabling it to regulate multiple biological processes, including the integration of signaling pathways (101). For example, research indicates that IGFBP2 might actively promote the movement of macrophages (102). These gene expression levels are down-regulated, indicating that the function of airway epithelial cells is impaired during asthma exacerbation.

The up-regulated genes in immune cells support the development, migration and activation of immune cells, enhance signal transduction, and facilitate the production of inflammatory cytokines and chemokines. Charcot-Leyden crystals (CLCs) are formed from the eosinophil granule protein galectin-10 (Gal10) and found in severe eosinophil-associated diseases like asthma. It is a biomarker of airway eosinophilia and it forms bipyramidal hexagonal crystals which can directly induce innate and type 2 immune response (103). Therapeutic antibodies with high affinity for Gal10 quickly dissolve crystals in patient mucus samples and decrease CLC-induced mucus production, inflammation, and IgE synthesis (104). There is anticipation about their potential impact on people with asthma. NFAM1 is a transmembrane receptor expressed in innate and adaptive immune cells. It is capable of inducing the activation of the calcium-dependent transcription factor NFAT, thereby promoting the expression of pro-inflammatory cytokines and chemokines across various cell types. Additionally, NFAM1 facilitates the activation of diverse immune cells, including T cells, macrophages, dendritic cells, and monocytes. It also plays a regulatory role in B lymphocyte development and signal transduction (105, 106). F13A1 is an important coagulation-related gene encoding factor XIII subunit A (FXIII-A). It is not only involved in blood coagulation, but also has a role in basic immunological functions as well (107). The cellular form of FXIII has been associated with phagocytic activities of macrophages, as well as the regulation of migration in monocyte-derived dendritic cells (108, 109). Esnault et al. also found that the RNA and protein level of F13A1 in BALF cells of asthma patients was significantly up-regulated after allergen exposure, and the expression level was positively correlated with the level of type 2 immune response. Combined with our analysis results, F13A1 in BALF of asthma patients mainly came from immune cells. It may also play a role in the acute exacerbation of asthma, but the exact mechanism is unclear (110, 111). These genes are up-regulated, indicating a dysregulated immune response in asthma exacerbation.

Apparently, the downregulation of key regulatory genes in epithelial cells and the enhancement of the immune related signaling pathways, particularly TGF-β signaling, which is a marker of epithelial-mesenchymal transition (EMT), suggest a loss of epithelial integrity and epithelial-mesenchymal balance in patients with asthma. Consistent with us, research showed that asthmatic samples exhibited stronger reactions to TGF-β compared to healthy controls, with more TGF-β-responsive basal cells and a decrease in epithelial markers alongside an increase in mesenchymal cell markers (112). Dysregulation of EMT in asthma could permit transformed cells to migrate into the airway submucosa and impair the wound healing ability of epithelial cells (113, 114). Inflammation and remodeling occur concurrently and promote each other, thereby maintaining asthma-associated pathology (113). Moreover, it is important to note that functional enrichment analysis of epithelial cells revealed upregulated immune signaling pathways, implying that airway epithelial cells also play a crucial role in regulating immune responses. For example, in addition to iron homeostasis, inflammation response was also involved in the differentiation of airway epithelial cells (**Supplementary Figure S2D**). The processes depend on the activation of the immune cells. In details, pattern recognition receptors (PRRs) on the surface of epithelial cells enable them to react to different external stimuli by generating chemokines and cytokines (115). This subsequently triggers the activation of DCs, which move to the draining nodes to encourage Th2 development, and activates innate immune cells (eosinophil, mast cells, et al.) that are drawn to the airways, leading to the production of numerous mediators that contribute to airway inflammation (116). To conclude, asthma is characterized as a chronic inflammation of the airways, in which airway epithelial cells and immune cells participate in and interact with each other (117). The airway epithelium serves not only as a passive shield against harmful external substances but also plays a crucial role in modulating the immune system with potentially significant contributions to asthma pathogenesis (115). Consequently, the interconnection of epithelial and immune cells may contribute to the persistence of asthma symptoms, and targeting only one factor in treatment might not offer enough clinical enhancement (113).

Finally, we performed validation in asthma models to elucidate the specific roles of these key regulated genes in disease progression. Both airway inflammation and mucous hypersecretion characteristics were presented in the asthma model induced by HDM stimulation. The pathological findings and qRT-PCR results indicate the successful establishment of the asthma model. The reliability of 9 key regulatory genes (TUBA1A, ICAM4, SCEL, TMPRSS11A, TMPRSS11B, IGFBP2, CLC, NFAM1, and F13A1) were supported by qRT-PCR verification.

Some limitations need to be elucidated in our study. First, our data may have variations introduced by differences in the sequencing depth of two GEO datasets, BALF sampling methods, and individual reactions to allergens. Second, our study did not employ verification using the asthma exacerbation model to enhance results credibility, only the asthma model was utilized, as there is no recognized method for modeling acute exacerbation of asthma, which complicates confirmation of success (116). Last, due to insufficient cell numbers in the BALF of mice for adequate RNA extraction for qRT-PCR, we verified key regulatory genes in epithelial and immune cells using lung tissue. The cell composition in the lung tissue of mice was complicated, which may cause interference with the results. Further clarifying the roles of these key regulated genes is a topic that we will explore in the future.

## 5 Conclusions

In summary, we revealed the multiple characteristics of functional enrichment, differentiate dynamics, cell-cell communication, and metabolic changes in epithelial and immune cells of asthma exacerbation at the single-cell level. Furthermore, we pinpointed 9 key regulatory genes (TUBA1A, ICAM4, SCEL, TMPRSS11A, TMPRSS11B, IGFBP2, CLC, NFAM1, and F13A1) in BALF of asthma by integrating scRNA-seq and bulk RNA-seq data. Our research elucidates cellular changes and intercellular interactions in asthma airways, offering new insights into the cellular and molecular mechanisms of asthma pathogenesis and progression, and potentially aiding in the development of more effective therapeutic interventions.

## Data Availability

The datasets presented in this study can be found in online repositories. The names of the repository/repositories and accession number(s) can be found in the article/Materials and methods.

## Supplementary material

Supplementary Figure 1 ScRNA-seq analysis and cell-type assignment strategy for the assignment of epithelial and immune cells. **(A, B)** UMAP plots showing a comparison of data quality before (left) and after (right) integration of cell clusters from all samples. color-coded by the sample ID. A1-A4, 4 samples in A group; C1-C4, 4 samples in C group. **(C)** Dot plot of canonical cell type marker genes for each cluster. **(D)** UMAP representation of epithelial and immune cell clusters from all samples. **(E)** Feature plots of the expression of canonical marker genes used for global cell-type assignment. **(F)** Bar plot of the cell type distribution in C and A groups. **(G)** Feature plots of the expression of canonical marker genes used for epithelial subsets assignment.

Supplementary Figure 2 Pseudotime analysis and cell-cell communications of epithelial cell populations. **(A, B)** Trajectory analysis reveals the differentiation process of epithelial populations **(A)** and pseudotime **(B)**. Trajectory directions were determined by a comprehensive consideration of biological prior and pseudotime analysis. **(C)** Heatmap displaying the dynamic expression of fate-determining genes which were obtained by BEAM analysis along the pseudotime trajectory, and these genes were clustered into 4 groups according to their expression pattern along the pseudotime. **(D)** GO enrichment analysis of differential related genes in heatmap. **(E)** The TF-mRNA regulatory network visualized by Cytoscape. Red represented mRNAs and blue represented TFs. **(F, G)** Circle plot showing the interaction numbers and strength between epithelial cell types comparing A with C. Blue lines indicated that the displayed communication is decreased in A, whereas red lines indicate that the displayed communication is increased in A compared with C. **(H)** Bar plots of the inferred interaction numbers (left) and strength (right) of epithelial cells in A and C groups. **(I)** Relative contribution of each ligand-receptor pair to the overall communication network. **(J)** Analysis of the interactions between C and A groups on the activity of overall (incoming and outcoming) signaling pathways.

Supplementary Figure 3 Metabolic analysis of epithelial cell populations. **(A)** Heatmap displaying the significantly different metabolic pathways between C and A groups. **(B-H)** Scatter plots comparing metabolic pathway activities between C and A groups for epithelial subsets.

Supplementary Figure 4 Cell-type assignment strategy for the assignment of immune subsets. **(A)** UMAP plot showing immune cell clusters from all samples, color-coded by Sample ID. A1-A4, 4 samples in A group; C1-C4, 4 samples in C group. **(B)** Feature plots of the expression of canonical marker genes used for immune subsets assignment.

Supplementary Figure 5 The lineage and characteristics of dendritic cells. **(A)** Stacked violin plot depicting distributions of DCs marker genes in each cell type cluster. **(B)** Volcano plot of DEGs for DCs and other cell types. The top 10 upregulated genes and top 10 downregulated genes were labeled according to the value of log2FC. **(C)** Trigram array plot demonstrating the GO pathway analysis of DEGs and the top 10 enrichment pathways. **(D)** KEGG analysis depicting the gene regulatory network between enrichment items and related genes. **(E)** The GSEA of C2 pathways of DCs and other cell types. NES, normalized enrichment score. C2: curated gene sets in MSigDB datasets.

Supplementary Figure 6 Metabolic analysis of immune cell populations. **(A)** Boxplot of the metabolic pathway activity of immune cell subsets in A. **(B)** Boxplot of the metabolic pathway activity of immune cell subsets in C. **(C)** Heatmap displaying the significantly different metabolic pathways of immune subsets between C and A. **(D)** UMAP plots showing the enrichment score of glutathione metabolism, purine metabolism, glycolysis and oxidative phosphorylation signal pathway.
