# Supplementary figures and images for: Epithelial and immune transcriptomic characteristics and possible regulatory mechanisms in asthma exacerbation: insights from integrated studies
Source: Front Immunol. 2025 Jan 23;16:1512053. doi: 10.3389/fimmu.2025.1512053 (PMC11798785; doi:10.3389/fimmu.2025.1512053)

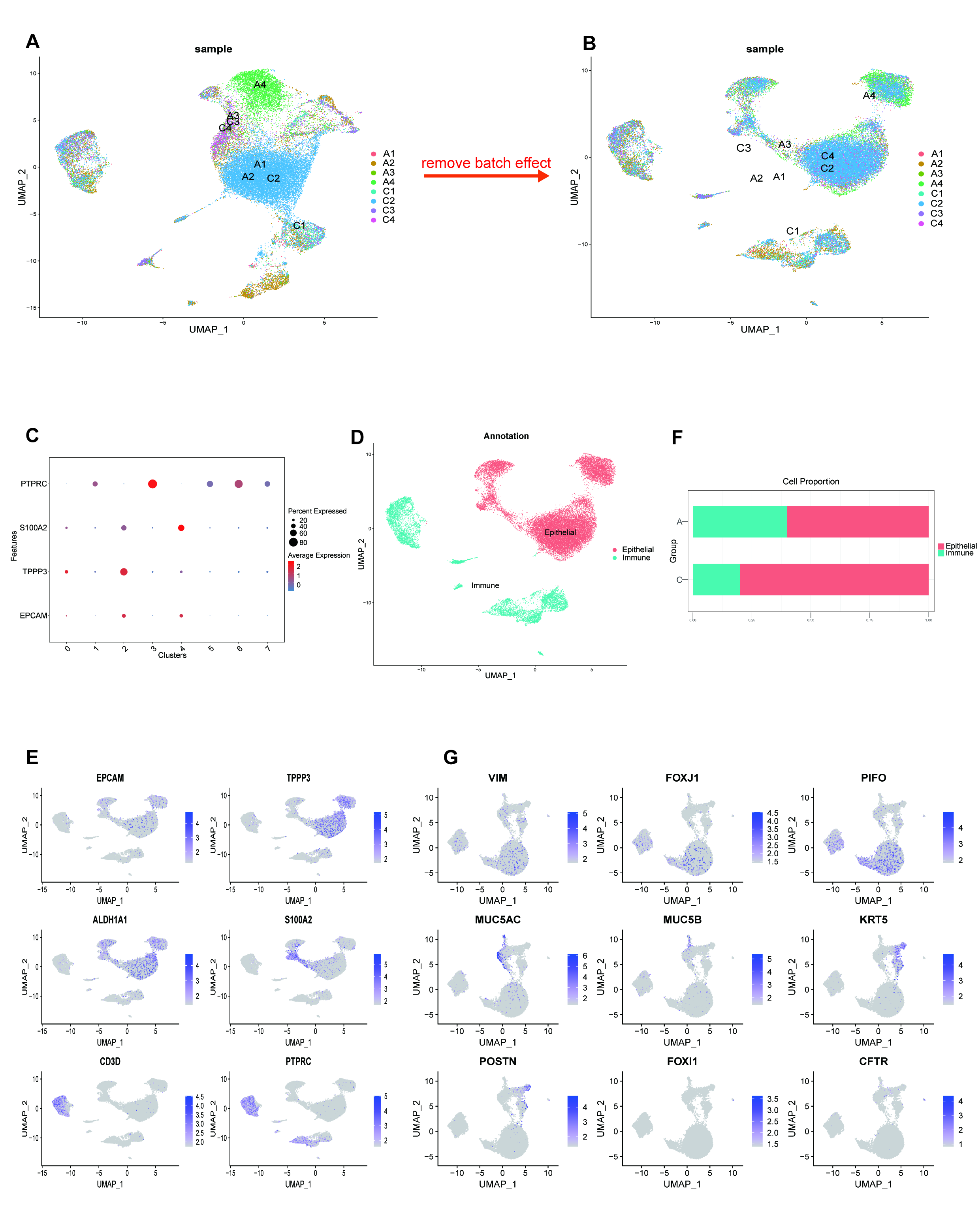

Supplement: Supplementary Figure 1 — ScRNA-seq analysis and cell-type assignment strategy for the assignment of epithelial and immune cells. (A, B) UMAP plots showing a comparison of data quality before (left) and after (right) integration of cell clusters from all samples. color-coded by the sample ID. A1-A4, 4 samples in A group; C1-C4, 4 samples in C group. (C) Dot plot of canonical cell type marker genes for each cluster. (D) UMAP representation of epithelial and immune cell clusters from all samples. (E) Feature plots of the expression of canonical marker genes used for global cell-type assignment. (F) Bar plot of the cell type distribution in C and A groups. (G) Feature plots of the expression of canonical marker genes used for epithelial subsets assignment. [file Image1.tif]

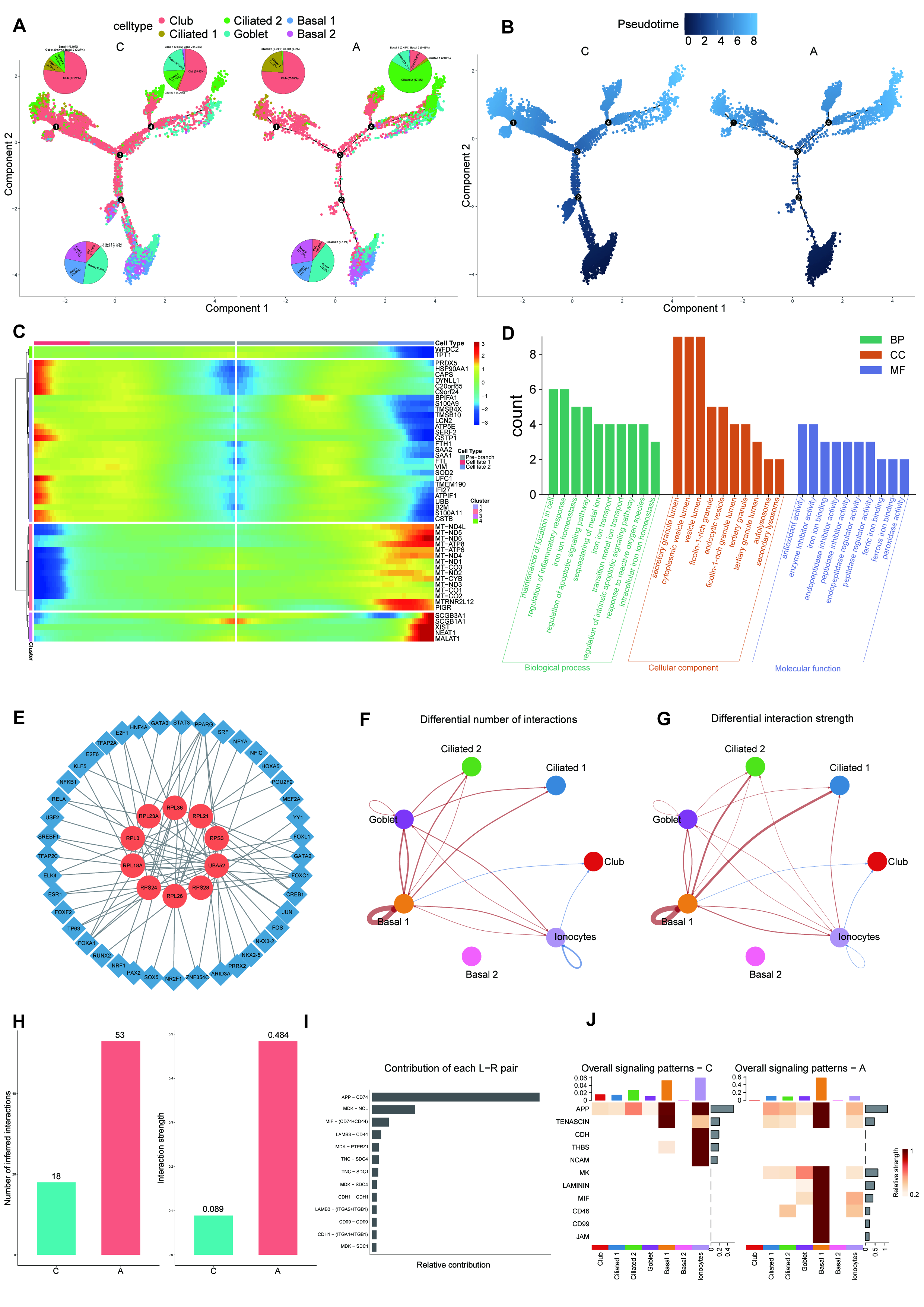

Supplement: Supplementary Figure 2 — Pseudotime analysis and cell-cell communications of epithelial cell populations. (A, B) Trajectory analysis reveals the differentiation process of epithelial populations (A) and pseudotime (B). Trajectory directions were determined by a comprehensive consideration of biological prior and pseudotime analysis. (C) Heatmap displaying the dynamic expression of fate-determining genes which were obtained by BEAM analysis along the pseudotime trajectory, and these genes were clustered into 4 groups according to their expression pattern along the pseudotime. (D) GO enrichment analysis of differential related genes in heatmap. (E) The TF-mRNA regulatory network visualized by Cytoscape. Red represented mRNAs and blue represented TFs. (F, G) Circle plot showing the interaction numbers and strength between epithelial cell types comparing A with C. Blue lines indicated that the displayed communication is decreased in A, whereas red lines indicate that the displayed communication is increased in A compared with C. (H) Bar plots of the inferred interaction numbers (left) and strength (right) of epithelial cells in A and C groups. (I) Relative contribution of each ligand-receptor pair to the overall communication network. (J) Analysis of the interactions between C and A groups on the activity of overall (incoming and outcoming) signaling pathways. [file Image2.tif]

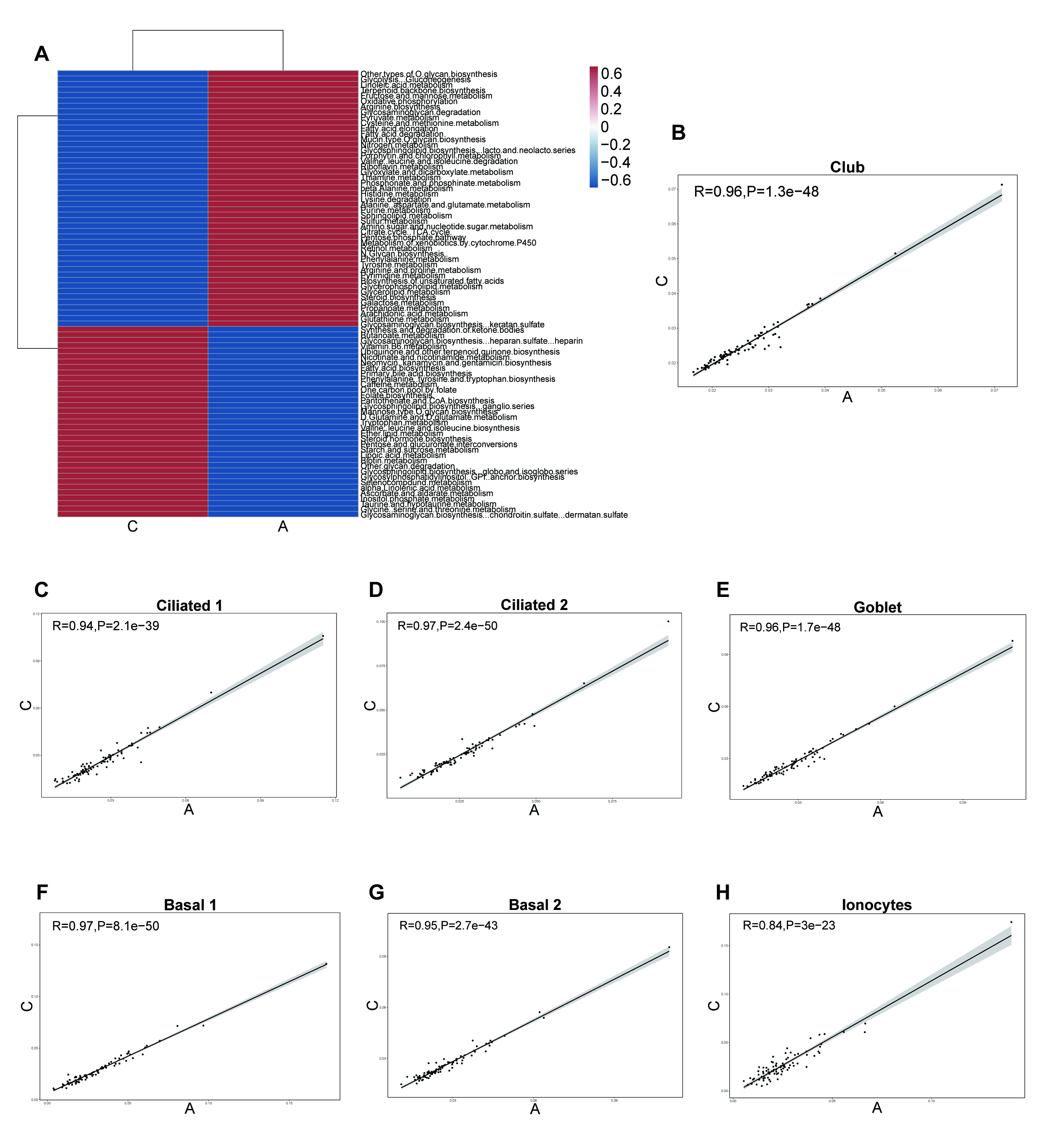

Supplement: Supplementary Figure 3 — Metabolic analysis of epithelial cell populations. (A) Heatmap displaying the significantly different metabolic pathways between C and A groups. (B-H) Scatter plots comparing metabolic pathway activities between C and A groups for epithelial subsets. [file Image3.tif]

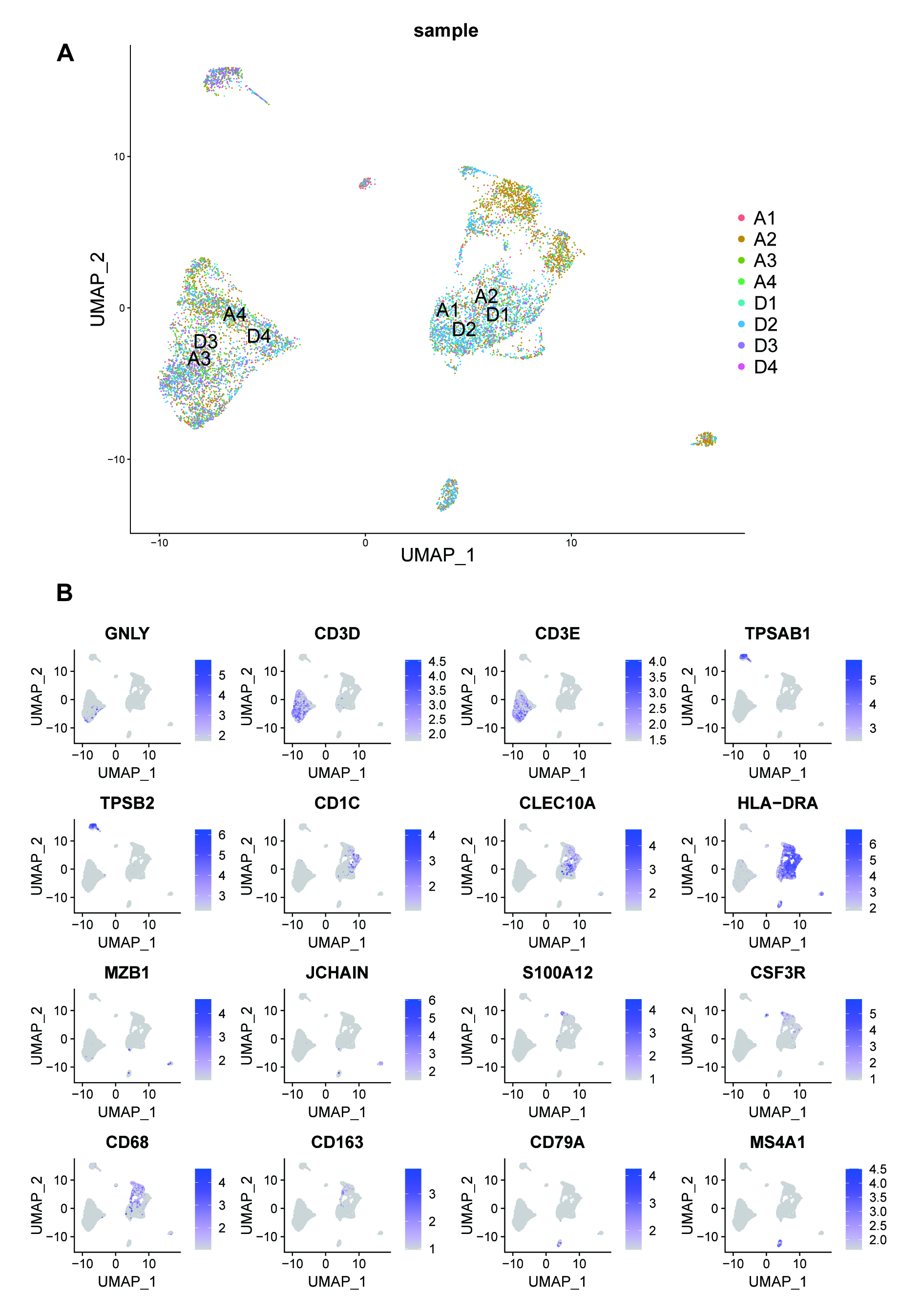

Supplement: Supplementary Figure 4 — Cell-type assignment strategy for the assignment of immune subsets. (A) UMAP plot showing immune cell clusters from all samples, color-coded by Sample ID. A1-A4, 4 samples in A group; C1-C4, 4 samples in C group. (B) Feature plots of the expression of canonical marker genes used for immune subsets assignment. [file Image4.tif]

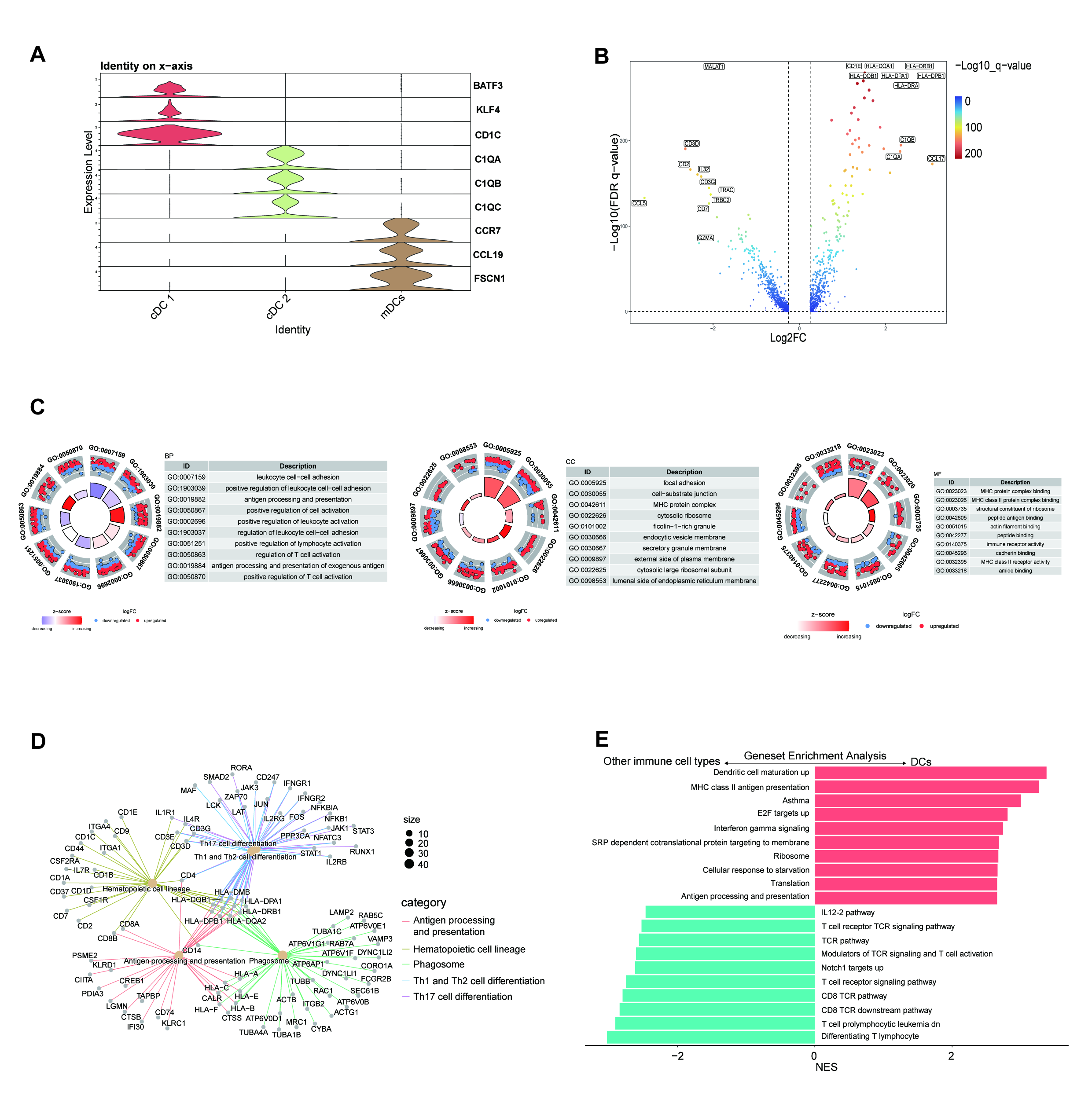

Supplement: Supplementary Figure 5 — The lineage and characteristics of dendritic cells. (A) Stacked violin plot depicting distributions of DCs marker genes in each cell type cluster. (B) Volcano plot of DEGs for DCs and other cell types. The top 10 upregulated genes and top 10 downregulated genes were labeled according to the value of log2FC. (C) Trigram array plot demonstrating the GO pathway analysis of DEGs and the top 10 enrichment pathways. (D) KEGG analysis depicting the gene regulatory network between enrichment items and related genes. (E) The GSEA of C2 pathways of DCs and other cell types. NES, normalized enrichment score. C2: curated gene sets in MSigDB datasets. [file Image5.tif]

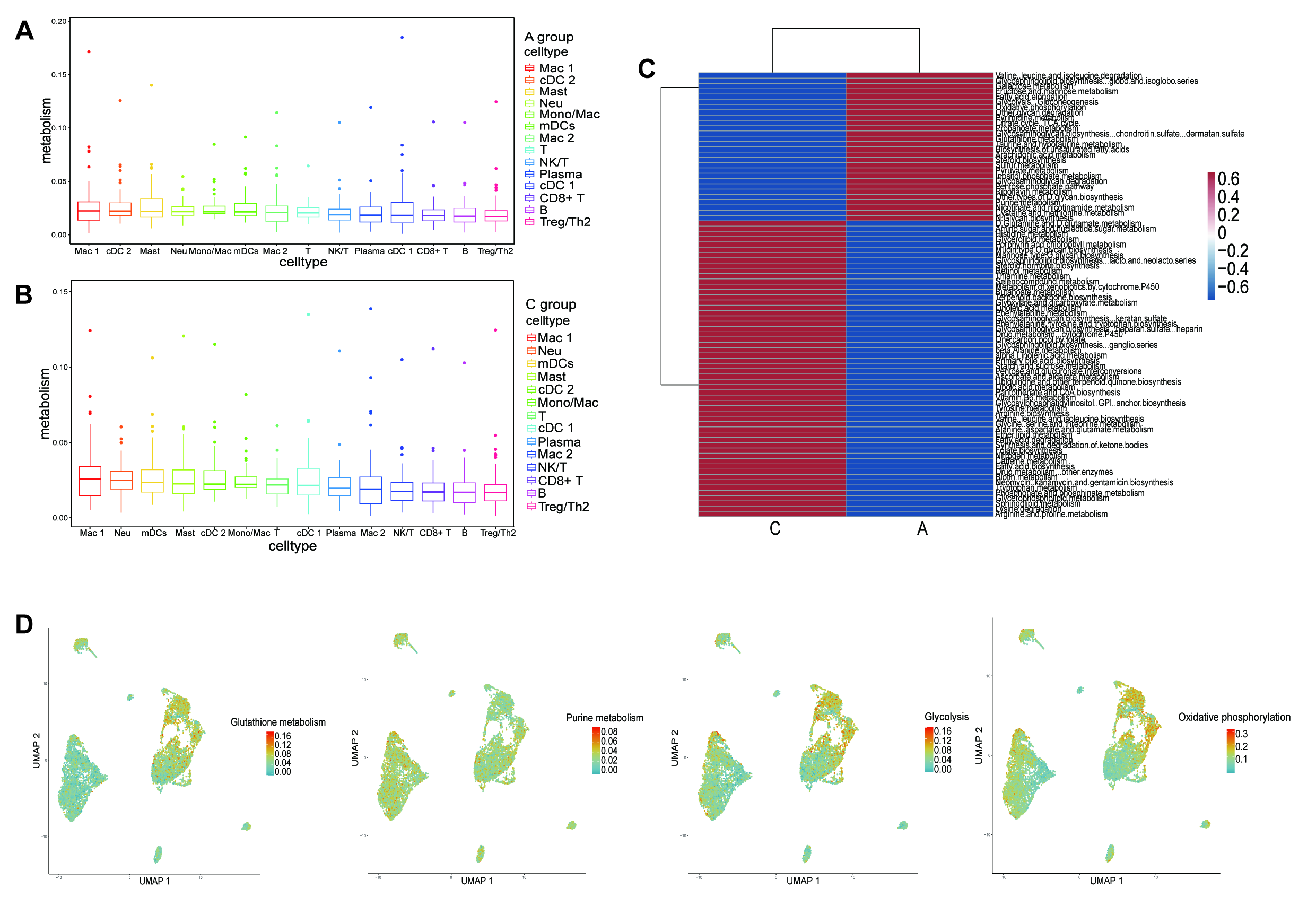

Supplement: Supplementary Figure 6 — Metabolic analysis of immune cell populations. (A) Boxplot of the metabolic pathway activity of immune cell subsets in A. (B) Boxplot of the metabolic pathway activity of immune cell subsets in C. (C) Heatmap displaying the significantly different metabolic pathways of immune subsets between C and A. (D) UMAP plots showing the enrichment score of glutathione metabolism, purine metabolism, glycolysis and oxidative phosphorylation signal pathway. [file Image6.tif]
