# Supplementary material for: Epithelial and immune transcriptomic characteristics and possible regulatory mechanisms in asthma exacerbation: insights from integrated studies
Source: Front Immunol. 2025 Jan 23;16:1512053. doi: 10.3389/fimmu.2025.1512053 (PMC11798785; doi:10.3389/fimmu.2025.1512053)
Supplement: Supplementary file 7 [file Table1.docx]

| **Table S1** The primers sequence for qRT-PCR | | |
| --- | --- | --- |
| Species | Primer name | Sequence（5'-3') |
| human | β-actin-F | AGAGCTACGAGCTGCCTGAC |
| human | β-actin-R | AGCACTGTGTTGGCGTACAG |
| human | IL25-F | TGAGGGAGCGACCCAGATTA |
| human | IL25-R | GCCAAGAATGCAACCACCTG |
| human | IL33-F | ATGAATCAGGTGACGGTGTTGAT |
| human | IL33-R | TCCACAGAGTGTTCCTTGTTGTT |
| human | TSLP-F | AATCGGCCACATTGCCTTAC |
| human | TSLP-R | CATGGCGAACATTTCTTTGG |
| human | POSTN-F | AGGATGTGACGGTGACAGTATAA |
| human | POSTN-R | CAGGTGCCAGCAAAGTGTATTC |
| human | IGFBP2-F | ACAACCTCAAACAGTGCAAGATG |
| human | IGFBP2-R | CCTCCTGCTGCTCATTGTAGAA |
| human | TMPRSS11A-F | CTTGGCAAGCTTCCCTTCAGTAT |
| human | TMPRSS11A-R | ACAACAGCAATGTCGTACTCTCT |
| human | TUBA1A-F | CTCTTCCACCCTGAGCAACTTAT |
| human | TUBA1A-R | TCAACTGAGAGACGTTCCATGAG |
| human | SCEL-F | CAGCATGTCCAATGTTACCTTGAG |
| human | SCEL-R | TTGAGCACCACCCTACCGTAATT |
| human | ICAM4-F | CATTACACTGATGCTCGCTTGGA |
| human | ICAM4-R | CCTTTACGCCTGGGACTTCATAG |
| human | TMPRSS11B-F | GCCTAATGCCAATGGTTCAAATG |
| human | TMPRSS11B-R | GCCTTGCTGATTTCCATGAGTTT |
| mouse | β-actin-F | CTAGGCGGACTGTTACTGAGC |
| mouse | β-actin-R | ATGTTTGCTCCAACCAACTGC |
| mouse | Ccl11-F | AGATGCACCCTGAAAGCCATAGT |
| mouse | Ccl11-R | GCTCTGGGTTAGTGTCAAGAGAG |
| mouse | Ccl24-F | CATCTTGCTGCACGTCCTTTATT |
| mouse | Ccl24-R | TTATGGCCCTTCTTGGTGATGAA |
| mouse | Ccl26-F | TGTTCTTCGATTTGGGTCTCCTT |
| mouse | Ccl26-R | GCTCTTGTCGGTGAACTTATAGC |
| mouse | Muc5ac-F | GTTCCATAGCCTCCTCTTGTTCT |
| mouse | Muc5ac-R | TCATGGCAGAGCTTGAAGGTATT |
| mouse | Nfam1-F | GAATCTGTCTACACGTCCCTACA |
| mouse | Nfam1-R | CAAGTCTCGGAGCCTGTAGAATT |
| mouse | F13a1-F | GGTGAATGCCAAGGATGATGAAG |
| mouse | F13a1-R | TGGGCCGAGAAGTAATTGGTAAT |
| mouse | Igfbp2-F | AAGCAGTGCAAGATGTCTCTGAA |
| mouse | Igfbp2-R | AGTCTCCTGCTGCTCGTTGTA |
| mouse | Tmprss11a-F | CTCTGCATCTTTCCCACCAAATT |
| mouse | Tmprss11a-R | CCTTCCAGAAATCCAGCACAGAA |
| mouse | Tuba1a-F | CCGCGAAGCAGCAACCAT |
| mouse | Tuba1a-R | GCCATGTTCCAGGCAGTAGA |
| mouse | Scel-F | GGCCAGGACCTAGAAAGCTTAAC |
| mouse | Scel-R | CTTGTGGACTCCTTGAACAGACT |
| mouse | Icam4-F | TGCGGCAGGGAAAGATAGTTAAT |
| mouse | Icam4-R | GCCGTTTGTAAGCAGTGATCCT |
| mouse | Tmprss11b-F | GAAGCTTATGGAGGCAGGATAGT |
| mouse | Tmprss11b-R | CTTGTTGACTCTGCCACATTCAT |
